# Single-cell transcriptomic analysis of chondrocytes in cartilage and pathogenesis of osteoarthritis

**DOI:** 10.1016/j.gendis.2024.101241

**Published:** 2024-02-02

**Authors:** Changyuan Huang, Bin Zeng, Bo Zhou, Guanming Chen, Qi Zhang, Wenhong Hou, Guozhi Xiao, Li Duan, Ni Hong, Wenfei Jin

**Affiliations:** a Harbin Institute of Technology, Harbin, Heilongjiang 150001, China; b School of Life Sciences, Southern University of Science and Technology, Shenzhen, Guangdong 518055, China; c Department of Orthopedics, Shenzhen Intelligent Orthopaedics and Biomedical Innovation Platform, Guangdong Artificial Intelligence Biomedical Innovation Platform, Shenzhen Second People's Hospital, The First Affiliated Hospital, Shenzhen University, Shenzhen, Guangdong 518035, China; d Graduate School, Guangxi University of Chinese Medicine, Nanning, Guangxi 53020, China; e The First Dongguan Affiliated Hospital, Guangdong Medical University, Dongguan, Guangdong 523710, China; f Department of Biochemistry, School of Medicine, Southern University of Science and Technology, Guangdong Provincial Key Laboratory of Cell Microenvironment and Disease Research, Shenzhen, Guangdong 518055, China; g CAS Key Laboratory of Computational Biology, Shanghai Institute of Nutrition and Health, University of Chinese Academy of Sciences, Chinese Academy of Sciences, Shanghai 200031, China

**Keywords:** Cell heterogeneity, Chondrocyte apoptosis, Chondrocyte differentiation, Chondrocyte fibrosis, Osteoarthritis, Single-cell RNA-seq

## Abstract

Chondrocyte is considered the only cell type in cartilage. However, the cell heterogeneity of chondrocytes in human articular cartilage is still not well defined, which hinders our understanding of the pathogenesis of osteoarthritis (OA). Here, we constructed a single-cell transcriptomic atlas of chondrocytes in healthy cartilage and identified nine chondrocyte subsets including homeostatic chondrocytes, proliferate fibrochondrocytes, and hypertrophic chondrocytes (HTC). Interestingly, we identified two distinct HTC subpopulations, among which HTC-1 specifically expressed genes associated with apoptosis and programmed cell death. We identified two main trajectories of chondrocytes, one of which differentiates into fibrochondrocytes, while the other terminates in apoptosis. Comparison of chondrocyte subsets between healthy and OA cartilage showed that proliferate fibrochondrocytes and HTC-1 expanded in OA patients, whereas homeostatic chondrocytes decreased. Interestingly, we discovered an OA-specific proliferate fibrochondrocyte subset that may contribute to the development of OA via inflammation. In summary, this study significantly enhanced our understanding of cell heterogeneity of chondrocytes in articular cartilage and provides insight into the pathogenesis of OA.

## Introduction

Articular cartilage is a specialized connective tissue located on the surface of the synovial joint and plays an important role in lubrication and weight-bearing.^1^ With aging, progressive degeneration of articular cartilage leads to joint pain and dysfunction, namely osteoarthritis (OA). OA is the most common type of chronic musculoskeletal disease which is characterized by degeneration of articular cartilage, fibrosis of articular cartilage, formation of osteophyte, inflammation of synovium, and loss of mobility. OA has affected 7% of the global population, or more than 500 million people worldwide.^2^^,^^3^ Clinically, the knee joint is the most common site of OA, followed by the hand and hip joints.^4^ Furthermore, the global prevalence of OA is higher in women and increases with age, with 10% of men and 18% of women over 60 years old being affected.^5^ However, there are no effective therapies except for joint replacement in the late stage of OA, because the molecular mechanisms underlying the progression of OA remain largely unknown.

Chondrocyte is considered the only cell type in cartilage, which secretes growth factors and enzymes to regulate extracellular matrix synthesis.^6^^,^^7^ Chondrocytes are derived from mesenchymal stromal cells which differentiate into chondroprogenitors and then into chondrocytes.^8^^,^^9^ After chondrogenesis, chondrocytes remain as resting cells to form articular cartilage or exhibit a life cycle of proliferation, maturation, hypertrophy, and apoptosis.^10^^,^^11^ The degeneration of articular cartilage prompts the release of cytokines from damaged cartilage, thus triggering synovial fibrosis.^12^^,^^13^ Fibrosis is thought to be a prominent and consequential hallmark of OA, which includes fibrosis of synovial and generation of fibrocartilage.^12^ Although it is well known that cartilage is composed of chondrocytes, the cell heterogeneity of chondrocytes in human articular cartilage is not well defined.

Single-cell sequencing, in particular single-cell RNA sequencing (scRNA-seq), is a powerful tool to study cell heterogeneity, which has identified various cell types and provided insights into physiological and pathological processes of diseases.^14^, ^15^, ^16^, ^17^, ^18^ Recently, several studies used scRNA-seq to explore the cell heterogeneity of chondrocytes in cartilage from OA or other joint disease patients.^19^, ^20^, ^21^, ^22^, ^23^ Ji et al identified seven chondrocyte subsets in human OA cartilage, including proliferative chondrocytes (ProC), prehypertrophic chondrocytes (PreHTC), and hypertrophic chondrocytes (HTC). Furthermore, they identified chondrocyte subsets and their specific genes and found a potential transition among ProC, PreHTC, and HTC.^19^ Sun et al^20^ constructed a chondrocyte atlas in the healthy and degenerated meniscus, in which most chondrocyte subsets were consistent with that reported in Ji et al.^19^ Whereas Fu et al^22^ constructed a chondrocyte atlas and named chondrocyte subsets based on their significant enriched gene ontology (GO). Lv et al^23^ identified ferroptotic chondrocytes based on molecular characteristics and their markers in OA patients. This study also found that *TRPV1* protected chondrocytes from ferroptosis and could be an anti-ferroptotic target. Swahn et al^24^ found a senescent chondrocyte subset with ZEB1 as the main regulator that promoted OA in cartilage and meniscus. Although these studies identified chondrocyte subsets in human cartilage, these results are not well consistent, and dynamic processes of chondrocyte subsets in the progression of OA are not clear.

In this study, we performed scRNA-seq on chondrocytes from cartilage to better elucidate the cell heterogeneity of chondrocytes in human healthy cartilage and OA cartilage. We identified chondrocyte subsets using pre-defined markers and constructed a single-cell transcriptomics atlas of cartilage chondrocytes. The trajectory analysis was used to infer the potential transition and dynamics among chondrocyte subsets. We further compared the single-cell landscape between healthy cartilage and OA cartilage to reveal the distinct landscape of OA cartilage. These results offer a better understanding of the chondrocyte heterogeneity and provide a deeper insight into the pathogenetic mechanisms of OA.

## Materials and methods

### Collection and culture of chondrocytes

Human joint cartilage tissues were collected from Shenzhen Second People's Hospital. The healthy donor signed informed consent approved by the Institutional Review Board (IRB) of Shenzhen Second People's Hospital (ID: 20201109001-FS01). The cartilage was isolated from knee joints of the healthy human donor and OA patients and cultured following previous studies.^9^^,^^19^ In brief, cartilage was immediately put in physiological saline containing heparin anticoagulant at 4 °C after collection, which was further processed within 6 hours. Then the cartilage was cut into pieces (1 mm^3^) and digested with 0.2% collagenase in high-glucose Dulbecco's modified Eagle's medium (Gibco, Australian) containing 10% fetal bovine serum (Gibco, Australian) and 10 μg/L basic fibroblast growth factor (Gibco, Australian). Following overnight incubation at 37 °C with 5% CO_2_, cells were collected by centrifugation, washed twice, resuspended in high-glucose Dulbecco's modified Eagle's medium supplemented with 10% fetal bovine serum and 10 μg/L basic fibroblast growth factor, plated in a culture flask, and allowed to attach for three days. Nonadherent cells were removed after a seven-day culture and the medium was replaced. Medium replacement was carried out every 72 hours until the cells reached an 80% confluent layer. Cells were harvested with 0.25% (w/v) trypsin plus 0.02% (w/v) EDTA (Hyclone, USA) and subcultured at a density of 1000 cells/cm^2^.

### Single-cell scRNA-seq library preparation and sequencing and public data

Chondrocytes were isolated from the cultured cells and subjected to fluorescence-activated cell sorting using the BD FACSAria II instrument (BD Biosciences) to eliminate nonviable cells. scRNA-seq was conducted using the 10X genomics platform. Chromium Single Cell 3'Gel Bead and Library Kit (P/N 120237, 120236, 120262, 10X Genomics) were used following protocol. Each channel accommodated approximately 15,000 cells. Sequencing libraries were subsequently loaded on the Illumina NovaSeq 6000 platform using paired-end kits. We further obtained scRNA-seq data of articular cartilage from Swahn et al (GSE220243),^24^ namely Sw_data.

### Pre-processing of scRNA-seq data

The raw data were processed following our previous studies.^9^^,^^18^^,^^25^ In detail, the raw sequencing data was transformed into FASTQ format using the Illumina bcl2fastq software. To align the reads and demultiplex the barcodes, we employed Cell Ranger V2.2.0 from 10X Genomics, aligning the reads to the hg38 reference genome. The resulting digital gene expression matrices underwent preprocessing and filtering using the R packages scran and scater.^26^ Cells surpassing the expression threshold of 4000 genes (potentially indicating doublets), falling below 200 expressed genes (suggesting low-quality libraries), or exhibiting mitochondrial unique molecular index counts exceeding 10% (possibly indicative of cell fragments and debris) were excluded from subsequent analysis. Additionally, we utilized Scrublet^27^ to identify potential doublets, calculating a doublet score for each cell and determining the threshold based on the default parameters of the bimodal distribution. We set the expected doublet rate at 0.08, and cells predicted to be doublets or with a doubletScore parameter exceeding 0.25 were removed from consideration. After implementing rigorous quality control measures, the healthy cartilage retained a total of 13,363 cells, while OA#1 and OA#2 retained 8808 cells and 12,770 cells, respectively. After quality control of Sw_data, six normal cartilage samples retained 8505, 7183, 3601, 6519, 6243, and 7214 cells, while six OA samples retained 4389, 4458, 7060, 5362, 4944, and 5468 cells, respectively.

### Dimension reduction and visualization of scRNA-seq data

Seurat^28^ package was used for performing scRNA-seq data analysis, including data integration, normalization, dimension reduction, and cell clustering, following our previous studies.^9^^,^^18^^,^^25^ We implemented a gene-wise scaling approach to set the mean and variance of each gene across cells to 0 and 1, respectively, thus preventing highly expressed genes from dominating subsequent analyses. The scaled expression data was then employed to identify highly variable genes, which were subsequently utilized for dimension reduction. The UMAP algorithm was applied for the visualization of the scRNA-seq data.^29^

### Identification of cluster-specific genes and differentially expressed genes

We assigned annotations to each cell cluster based on the highly expressed genes specific to that particular population, as well as the established marker genes unique to each population. By employing the Wilcoxon Rank-Sum test, we compared the gene expressions within each investigated cluster to those of the remaining clusters. Genes exhibiting significantly higher expression levels within the investigated cluster were identified as cluster-specific genes. Furthermore, we performed the Wilcoxon Rank-Sum test to determine the differentially expressed genes between any two clusters. To ascertain statistical significance, a minimum log2(fold change) threshold of 0.25 and an adjusted *P*-value of 0.01 were applied. Metascape was applied for the investigation of biological process enrichment.^30^

### Analysis of ligand–receptor interaction

To investigate the intricate network of cellular communication, we employed the CellChat package (version 1.6.1) for ligand–receptor interaction analysis.^31^ Leveraging the extensive ligand–receptor pair data available in CellChatDB, we evaluated the potential interactions among the different cell populations. Specifically, we focused on the datasets pertaining to “secreted signaling”, “ECM–receptor”, and “cell–cell contact” interactions. These selected datasets provided valuable insights into the intricate cell communication occurring between each cluster. We also used CellPhoneDB^32^ and iTalk^33^ to infer cell–cell interaction between chondrocyte subsets.

### Trajectory analysis

BAM files aligned using the Cell Ranger pipelines were initially sorted using SAMtools.^34^ Next, the Velocyto pipeline was used to count spliced and un-spliced reads and generate loom files.^35^ To compute gene-specific velocities, we utilized the scVelo Python package.^36^ Additionally, the projection clustered with metabolic genes was embedded with the velocity streams predicted by scVelo with the loom files. Finally, plots for the ratio of spliced and un-spliced, for the velocity and the expression of various individual genes were generated based on the velocity calculated by scVelo. To verify the robustness of our findings, we employed additional developmental trajectory inference algorithms, specifically partition-based graph abstraction (PAGA).^37^ For PAGA analysis, pseudotime was calculated using scanpy v1.4.3. Briefly, we followed the pipeline integrated into scVelo, employing the same projection generated by scVelo. We performed the prediction using the scv.pl.paga function in scVelo, setting the basis parameter as UMAP, the size as 50, the alpha as 0.3, the min_edge_width as 2, and the node_size_scale as 1.5. We also used monocle2^38^ to infer the trajectory of chondrocytes of Sw_data.

### Statistical analysis

The programming languages R and Python were employed for all statistical analyses and data visualizations. Wilcoxon Rank Sum test was used to identify the differentially expressed genes between two cell clusters. Bonferroni correction was applied for multiple testing.

## Results

### A single-cell transcriptomic atlas of chondrocytes in healthy human cartilage

**Figure 1:**
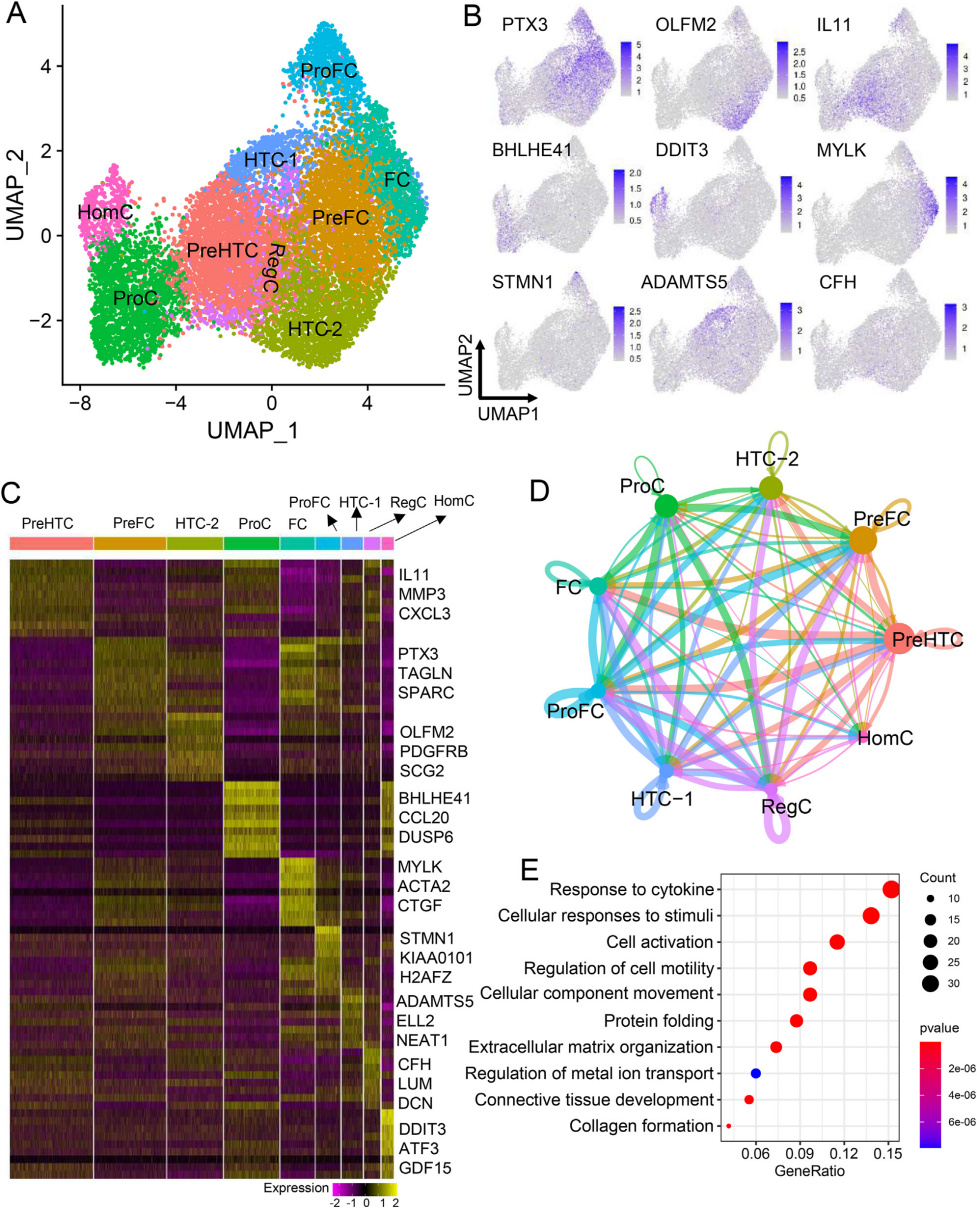
A single-cell transcriptomic atlas of chondrocytes in healthy human cartilage. **(A)** UMAP visualization of the 13,363 chondrocytes from healthy human cartilage. Color represents the chondrocyte subset. **(B)** UMAP visualization of the expression of representative marker genes for each chondrocyte subset. **(C)** The heatmap of chondrocyte subset-specific genes. **(D)** Cell–cell communication between chondrocyte subsets was analyzed by CellChat. The width and color of the line represent the strength of cell–cell interaction and signaling source, respectively. **(E)** Gene ontology (GO) enrichment of RegC-specific genes. HomC, homeostatic chondrocytes; PreHTC, prehypertrophic chondrocytes; ProC, proliferate chondrocytes; HTC, hypertrophic chondrocytes; ProFC, proliferate fibrochondrocytes; preFC, prefibrochondrocytes; FC, fibrochondrocytes; RegC, regulatory chondrocytes.

To reveal the cell heterogeneity of human chondrocytes, we conducted scRNA-seq on chondrocytes from healthy human knee cartilage. We obtained single-cell transcriptomes from 13,363 cells, with a median number of 10,606 detected unique molecular indexes and an average of 2753 detected genes per cell after quality control (Fig. S1A, B and Table S1). Unsupervised clustering of the chondrocytes resulted in a total of nine cell clusters (Fig. 1A). We annotated each cluster according to cluster-specific genes: (i) homeostatic chondrocytes (HomC) (*DDIT3*, *ATF3*, and *GDF15*), (ii) proliferative chondrocytes (ProC) (*BHLHE41*, *CCL20*, and *DUSP6*), (iii) prehypertrophic chondrocytes (PreHTC) (*IL11*, *MMP3*, and *CXCL3*), (iv) hypertrophic chondrocytes-1 (HTC-1) (*FMOD*, *EBF1*, *ADAMTS5*, *ELL2*, and *NEAT1*), (v) hypertrophic chondrocytes-2 (HTC-2) (*FMOD*, *EBF1*, *OLFM2*, *PDGFRB*, and *SCG2*), (vi) prefibrochondrocytes (PreFC) (*PTX3*, *TAGLN*, and *SPARC*), (vii) proliferate fibrochondrocytes (ProFC) (*STMN1*, *KIAA0101*, and *H2AFZ*), (viii) fibrochondrocytes (FC) (*MYLK*, *ACTA2*, and *CTGF*), and (ix) regulatory chondrocytes (RegC) (*CFH*, *LUM*, and *DCN*) (Fig. 1A–C; Fig. S1C–E). Among all chondrocyte subsets, PreHTC and PreFC were abundant and accounted for 22% and 19% of the total cells, respectively; while HomC and RegC were relatively rare and accounted for 3% and 4% of the total cells, respectively (Fig. S1F). We found ProFC expressed cell cycle genes including *STMN1*, *KIAA0101*, and *MCM3* (Fig. 1C; Fig. S1C). Further analysis showed that ProFC were mainly in the S phase of the cell cycle (Fig. S1G). Meanwhile, ProFC-specific genes enriched in the cell cycle, DNA replication, cell activation, and collagen formation (Fig. S2), strongly supporting that ProFC is in an active phase of the cell cycle. The GO terms enriched in the specifically expressed genes of each chondrocyte subset were consistent with its identity inferred by marker genes (Fig. S2).

We analyzed the crosstalk of ligand–receptor pairs to understand the cell-cell communication between chondrocyte subsets. We found HomC had the lowest self-interactions among all chondrocyte subsets based on three cell–cell interaction analysis methods (Fig. 1D; Fig. S3A, B). In particular, HomC sent out a few cell–cell interaction signals (Fig. S3C). These results potentially indicate that HomC is relatively resting and isolated. RegC has one of the strongest inter-subset interactions and self-interactions among all chondrocyte subsets (Fig. 1D). The GO enrichment analysis showed that RegC-specific genes were enriched in extracellular matrix organization, regulation of cellular component movement, regulation of cell motility, collagen formation, cellular responses to stimuli, connective tissue development, *etc*. (Fig. 1E). These results indicated that RegC played an important role in shaping cartilage microenvironment and regulation of chondrocyte movement and activation.

We used CellChat to identify cell–cell interaction signaling among chondrocyte subsets and the most significant cell–cell interaction signaling pathways included the COLLAGEN signaling pathway, FN1 signaling pathway, THBS signaling pathway, LAMININ signaling pathway, TENASCIN signaling pathway, and HSPG signaling pathway (Fig. S4A–F). These cell–cell interaction signaling pathways showed distinct patterns, indicating each pathway has its own feature and story. Taking the COLLAGEN signaling pathway as an example, FC displayed the strongest interaction with the other cell clusters, which indicates that the collagen metabolism in FC was more active than the other cell types (Fig. S4A, G, H).

### Differences between the two HTC subpopulations

**Figure 2:**
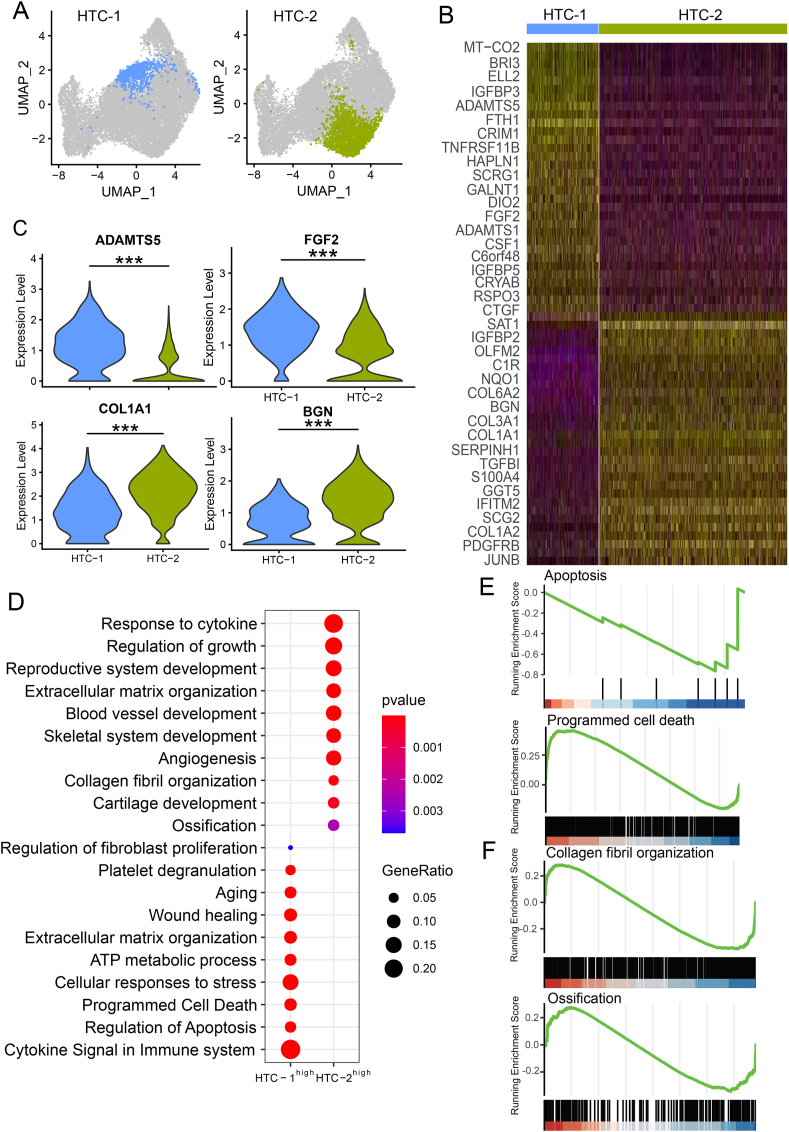
The different features of the two HTC populations. **(A)** Highlighting of the two HTC subpopulations on the UMAP plot of chondrocytes. **(B)** The heatmap of the expression level of differentially expressed genes (DEGs) between HTC-1 and HTC-2. **(C)** The violin plots showing the expression levels of representative DEGs between HTC-1 and HTC-2. **(D)** Gene ontology (GO) enrichment analysis of HTC-1-specific genes and HTC-2-specific genes. **(E)** Gene set enrichment analysis (GSEA) showed apoptosis and programmed cell death were associated with HTC-1-specific genes. **(F)** GSEA showed collagen fibril organization and ossification were associated with HTC-2-specific genes. HTC, hypertrophic chondrocytes.

We found two HTC subpopulations in human cartilage (Fig. 2A), and it is interesting to investigate the similarities and differences between the two HTC subpopulations. Although both HTC subpopulations highly expressed chondrocyte hypertrophic specific genes (Fig. S1D, E), we identified total 241 HTC-1-specific genes and 616 HTC-2-specific genes (Fig. 2B and Table S2); *ADAMTS5*^39^^,^^40^ and *FGF2*,^41^^,^^42^ which are associated with chondrocyte hypertrophy and cartilage degeneration, were expressed higher in HTC-1, while *COL1A1*^19^^,^^20^ and *BGN*,^43^^,^^44^ which are associated with fibrocartilage formation and collagen fibril organization, were expressed higher in HTC-2 (Fig. 2B, C). Moreover, GO enrichment analysis suggested that HTC-1-specific genes were enriched in the regulation of apoptosis, cellular responses to stress, and programmed cell death; while HTC-2-specific genes were enriched in skeletal system development, collagen fibril organization, and ossification (Fig. 2D–F), indicating the two HTC subpopulations have quite different functions.

### Pseudotime trajectories of chondrocytes and trajectory-associated genes

**Figure 3:**
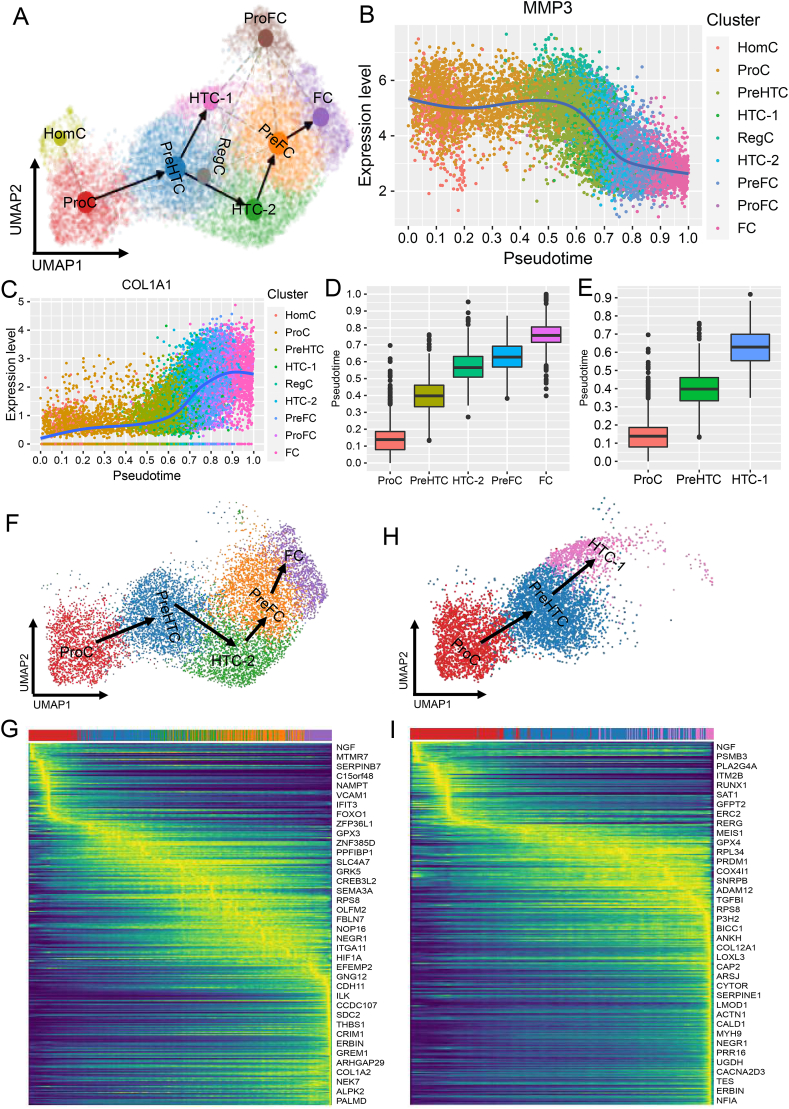
The pseudotime trajectories of chondrocytes and trajectory-associated genes. **(A)** The pseudotime trajectories of chondrocytes inferred by PAGA. **(B)** The expression of *MMP3* along pseudotime. **(C)** The expression of *COL1A1* along pseudotime. **(D)** Pseudotime score of ProC, PreHTC, HTC-2, PreFC, and FC in trajectory #1. **(E)** Pseudotime score of ProC, PreHTC, and HTC-1 in trajectory #2. **(F)** Trajectory #1 showed the progression of ProC, PreHTC, HTC-2, PreFC, and FC. **(G)** The dynamic gene expression along trajectory #1. **(H)** Trajectory #2 showed the progression of ProC, PreHTC, and HTC-1. **(I)** The dynamic gene expression along trajectory #2. PreHTC, prehypertrophic chondrocytes; ProC, proliferate chondrocytes; HTC, hypertrophic chondrocytes; preFC, prefibrochondrocytes; FC, fibrochondrocytes.

RNA velocity exploited the relative abundance of nascent (unspliced) and mature (spliced) mRNA to infer trajectory direction during dynamic processes.^35^^,^^36^ We calculated RNA velocity in each cell to infer the trajectories of chondrocytes using PAGA. We identified two main trajectories (trajectory #1: ProC → preHTC → HTC-2 → PreFC → FC, and trajectory #2: ProC → preHTC → HTC-1), which shared the starting point (Fig. 3A; Fig. S5, 6). The trajectories inferred by scVelo and monocle3 were similar to those inferred by PAGA (Fig. S5A, B). Interestingly, the expression of *MMP3* decreased along the pseudotime (Fig. 3B), consistent with recent reports that *MMP3* expressed in early chondrocyte development.^45^^,^^46^ The expression of *COL1A1* increased along the pseudotime (Fig. 3C), consistent with recent reports that *COL1A1* expressed in late chondrocyte development.^19^^,^^20^

We found that the pseudotime scores increased along either trajectory #1 or trajectory #2 (Fig. 3D, E). Trajectory #1, starting from ProC and ending up with FC, showed a process of chondrocyte proliferation, hypertrophy, and fibrosis (Fig. 3A, F), which was consistent with previous reports^19^^,^^47^ and Sw_data inferred by monocle2 (Fig. S6F, G). We identified hundreds of trajectory-coordinated genes with expression gradually changing along trajectory #1 that differentiated into FC (Fig. 3G). For example, *NGF*,^48^ *ITM2B*,^49^ and *RUNX1*,^50^ being reported associated with chondrocyte differentiation and proliferation, were highly expressed at the beginning of the trajectory (Fig. 3G). While *THBS1*,^51^ *COL1A2*,^52^ and *GREM1*,^53^ being reported associated with chondrocyte fibrosis, were highly expressed at the end of the trajectory (Fig. 3G; Fig. S5D). Trajectory #2 is the process of chondrocyte development, degradation, and apoptosis (Fig. 3A, H), which has not been reported at the single-cell level. The genes associated with chondrocyte degradation and apoptosis, such as *NFIA*,^54^ *SERPINE1*,^55^ and *CAP2*,^56^ were highly expressed in the later stage of trajectory #2 (Fig. 3I; Fig. S5E). Although it is reported that precisely regulated apoptosis plays an important role in the homeostasis of cartilage degradation *in vitro*,^47^^,^^57^^,^^58^ the trajectory of HTC apoptosis provides novel insight into the process of chondrocyte apoptosis and cartilage degradation.

### Systemic comparison of the single-cell landscape of chondrocytes between healthy individual and OA patients

**Figure 4:**
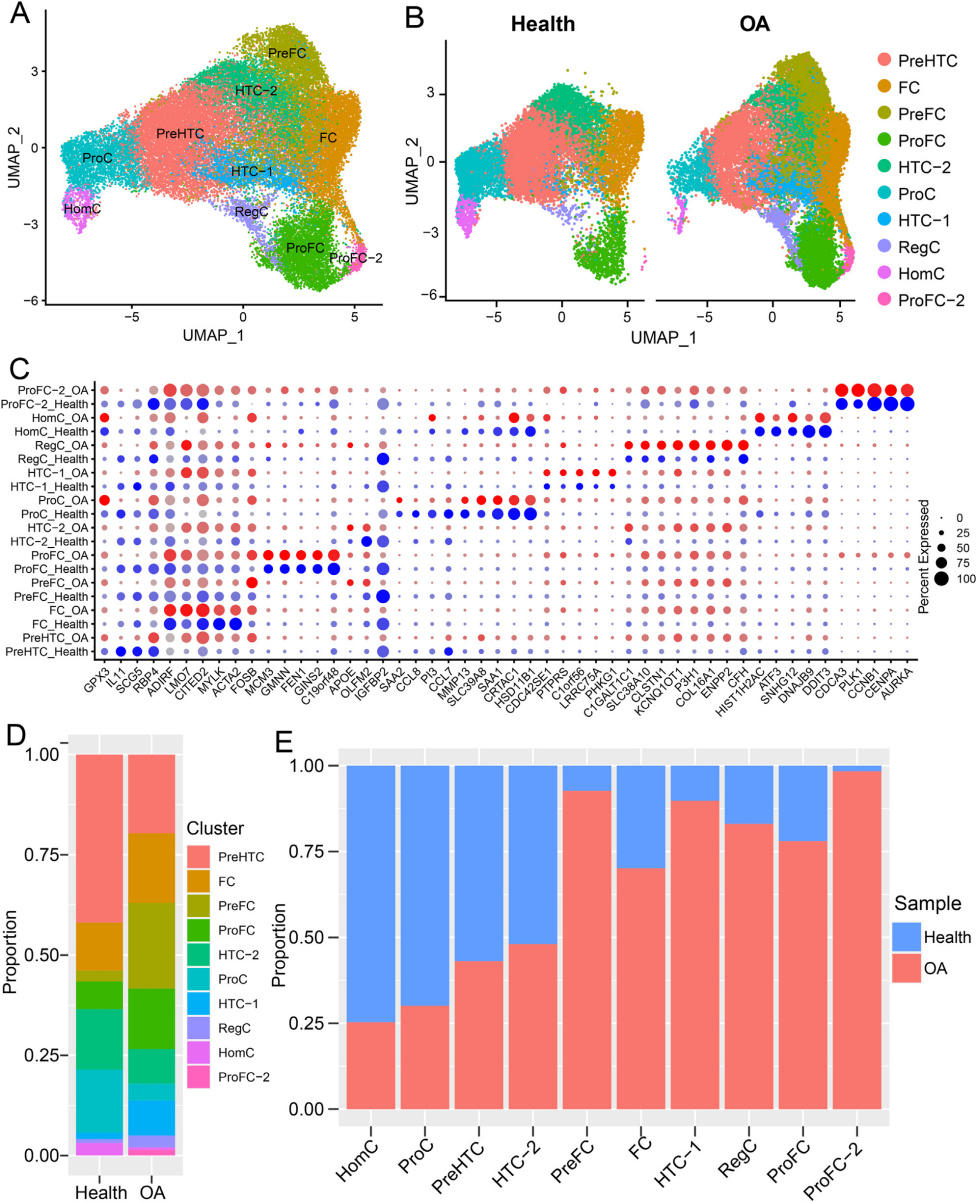
Comparison of the landscape of chondrocytes between healthy cartilage and OA cartilage. **(A)** UMAP visualization of 34,941 chondrocytes in healthy and OA cartilage. **(B)** UMAP visualization of chondrocytes in healthy cartilage (left) and OA cartilage (right). **(C)** Comparison of the expression of chondrocyte subset-specific genes between healthy cartilage and OA cartilage. Dot size and color intensity represent the fraction of cells expressing the genes and the average expression level, respectively. **(D)** Cell compositions of chondrocytes in healthy and OA cartilage. **(E)** The bar plot displaying the cell compositions of each chondrocyte subset based on cell sources. OA, osteoarthritis.

We conducted a comparative analysis of the single-cell landscape of chondrocytes between healthy cartilage and OA cartilage (Fig. 4A, B; Fig. S7A, B). After quality control, we had a total of 34,941 single-cell transcriptomes, comprising 13,363 cells from healthy individual and 21,578 cells from OA patients (Table S1). We identified ten chondrocyte subsets (Fig. 4A), nine of which were consistent with that in our constructed single-cell atlas (Fig. 1). PreHTC and PreFC were abundant, comprising 28% and 15% of the total cells, respectively, while ProFC and HomC were relatively scarce, accounting for only 1% and 2% of the total cells, respectively (Fig. S7F). Notably, we discovered a new chondrocyte subset, termed ProFC-2 that specifically expressed *CCNB1* and *MYLK* (Fig. 4A–C; Fig. S7E). Remarkably, ProFC-2 was exclusively present in OA cartilage, while the other clusters contained cells from both the healthy individual and OA patients (Fig. 4B). The expression of cluster-specific genes showed there were some genes expressed differently between healthy chondrocytes and OA chondrocytes (Fig. 4C). Furthermore, the proportions of PreFC, RegC, ProFC, and HTC-1 in OA patients were increased compared with those in the healthy individual, whereas the proportions of HomC, ProC, PreHTC, and HTC-2 in OA patients were decreased compared with those in the healthy individual (Fig. 4D, E; Fig. S7G, H). In particular, PreFC and HTC-1 were almost dominant by cells from OA patients, while HomC and ProC were almost dominant by cells from healthy individual (Fig. 4E; Fig. S7G, H), essentially consistent with independent analyses of Sw_data (Fig. S8A–J).

Both ProFC and ProFC-2 highly expressed cell cycle genes including *TOP2A* and *STMN1* (Fig. S9A, B). Gene set enrichment analysis of ProFC-specific genes and ProFC-2-specific genes revealed that both cell subsets enriched in the mitotic process (Fig. S9C, D), indicating that both ProFC and ProFC-2 are in an active state of cell proliferation. A total of 178 ProFC-specific genes and 329 ProFC-2-specific genes were identified by differential analysis (Fig. S9E and Table S3). ProFC-specific genes included *GINS2*, *HELLS*, and *MCM3*, while ProFC-2-specific genes included *CENPA*, *CDKN3*, and *AURKA* (Fig. S9B, F, G). GO enrichment analysis suggested that ProFC were enriched in extracellular matrix organization, skeletal system development, and cell cycle; while ProFC-2-specific genes were enriched in cytokine signaling, inflammatory response, and cellular responses to stimuli (Fig. S9H). Therefore, ProFC-2 might contribute to OA via inflammation since inflammation is thought to be associated with the development of OA.^59^

### Expanded cell populations in OA patients

**Figure 5:**
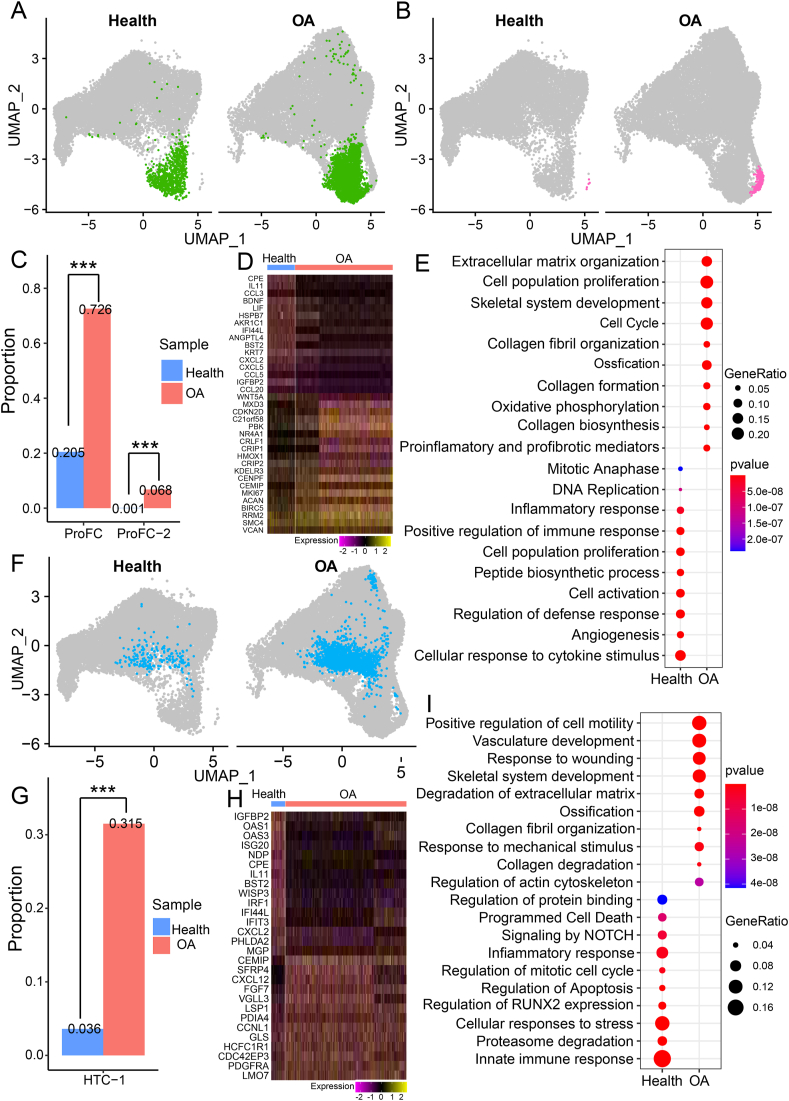
Expansion of ProFC, ProFC-2, and HTC-1 in OA cartilage and change of gene expression. **(A)** Highlighting of ProFC on UMAP plot of chondrocytes in healthy cartilage (left) and OA cartilage (right). **(B)** Highlighting of ProFC-2 on UMAP plot of chondrocytes in healthy cartilage (left) and OA cartilage (right). **(C)** The proportions of ProFC and ProFC-2 in healthy cartilage and OA cartilage. **(D)** The heatmap of differentially expressed genes (DEGs) between healthy cartilage and OA cartilage in ProFC. **(E)** Enrichment analysis of healthy specific genes and OA-specific genes in ProFC. **(F)** Highlighting of HTC-1 on UMAP plot in healthy cartilage (left) and OA cartilage (right). **(G)** The proportion of HTC-1 in healthy and OA cartilage. **(H)** The heatmap of the expression level of DEGs between healthy and OA cartilage in HTC-1. **(I)** Enrichment analysis of healthy specific genes and OA-specific genes in HTC-1. OA, osteoarthritis; HTC, hypertrophic chondrocytes; ProFC, proliferate fibrochondrocytes.

We identified three significantly expanded chondrocyte subpopulations in OA cartilage, namely ProFC, ProFC-2, and HTC-1. First, the proportion of ProFC in OA cartilage was significantly higher than that in healthy cartilage (Fig. 5A, C), indicating the increase of ProFC may be associated with or contribute to the occurrence and development of OA. Differential analysis of ProFC between healthy and OA cartilage identified 321 OA-specific genes (Fig. 5D and Table S4). These OA-specific genes include *CEMIP*,^60^^,^^61^ *ACAN*,^62^ and *HMOX1*^63^ which are associated with chondrocyte inflammation, degradation, or fibrosis. We also identified 437 healthy specific genes (Table S4) including *BDNF*, *IGFBP2*, and *WNT5A* (Fig. 5D). GO enrichment analysis of OA cartilage-specific genes in ProFC enriched in extracellular matrix organization, collagen fibril organization, and ossification, while healthy cartilage-specific genes in ProFC enriched in the cellular response to cytokine stimulus, cell activation, and cell population proliferation (Fig. 5E), indicating that ProFC in OA cartilage have increased extracellular matrix and collagen than in healthy cartilage. Intriguingly, ProFC-2 represented a small cell population predominantly in OA cartilage (Fig. 5B, C), implying that ProFC-2 have a unique effect on the occurrence and development of OA.

The proportion of HTC-1 in OA cartilage was significantly higher than that in healthy cartilage (Fig. 5F, G), which was consistent with the result of Sw_data (Fig. S8J). Differential analysis of HTC-1 between healthy and OA cartilage identified 230 OA-specific genes (Table S4) including *CEMIP*, *SFRP4*, and *CXCL12* (Fig. 5H). We also identified 333 healthy specific genes (Table S4) including *IGFBP2*, *WISP3*, and *IFIT3* (Fig. 5H). GO enrichment analysis of OA cartilage-specific genes in HTC-1 enriched in vasculature development, degradation of the extracellular matrix, and ossification, while healthy cartilage-specific genes in HTC-1 enriched in cellular response to stress, proteasome degradation, and regulation of apoptosis (Fig. 5I), implying that HTC-1 might be stimulated into apoptosis via degradation of the extracellular matrix, and the increase of HTC-1 might trigger OA.

### Decrease of HomC and changes in gene expression in OA patients

**Figure 6:**
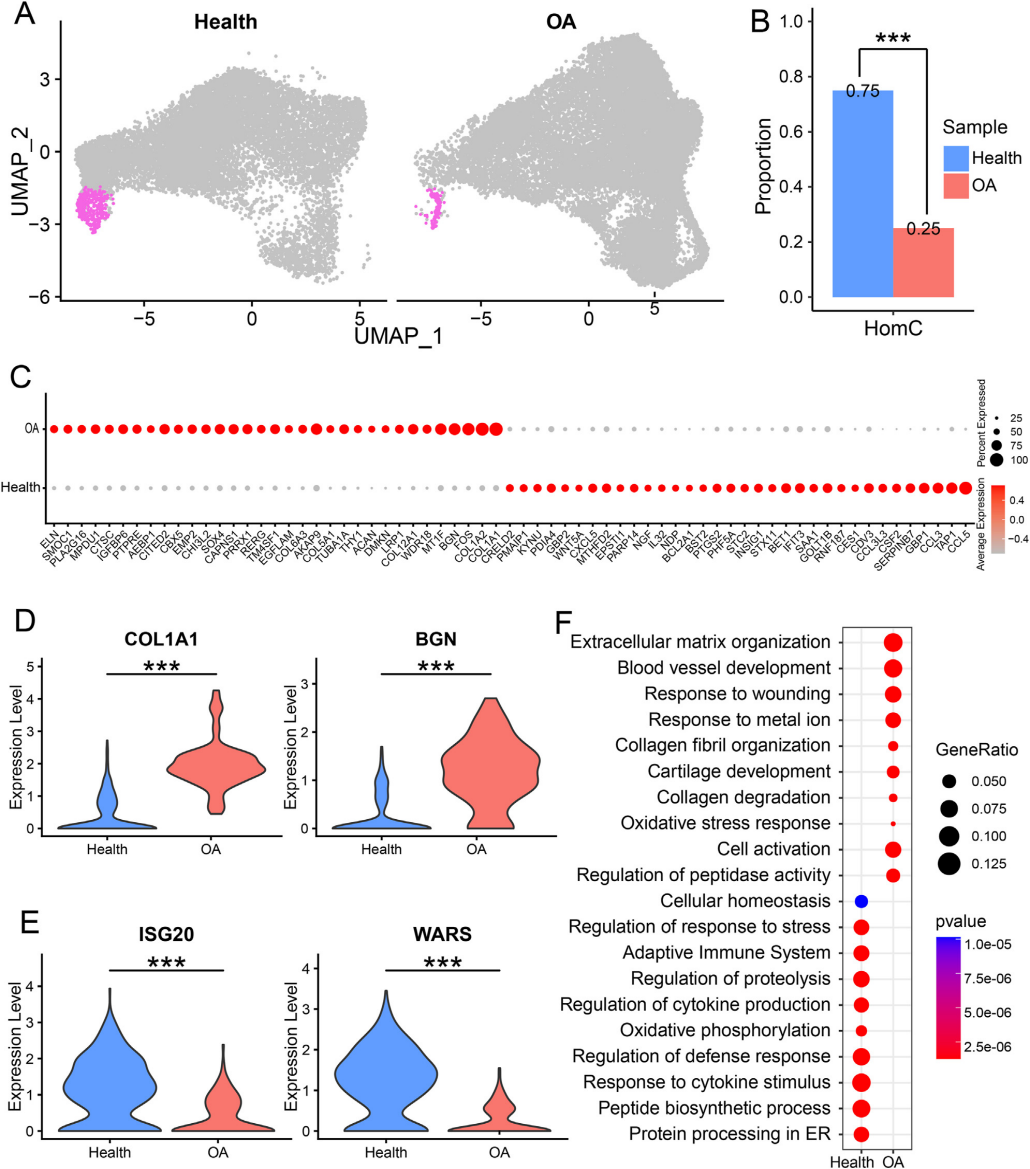
Reduction of HomC in OA cartilage and change of gene expression. **(A)** Highlighting of HomC on UMAP plot in healthy cartilage (left) and OA cartilage (right). **(B)** The proportion of HomC in healthy cartilage and OA cartilage. **(C)** The dotplot of healthy specific genes and OA-specific genes in HomC. **(D)** The violin plot of representative OA-specific genes. **(E)** The violin plot of representative healthy specific genes. **(F)** Enrichment analysis of healthy specific genes and OA-specific genes in HomC. OA, osteoarthritis; HomC, homeostatic chondrocytes.

Although we found several chondrocyte subsets expanded in OA cartilage, the proportion of HomC in OA cartilage was significantly lower than that in healthy cartilage (Fig. 6A, B), which was consistent with independent analyses of Sw_data (Fig. S8I). HomC have been reported for their protective role in preventing cartilage degeneration and exhibit high expression of human circadian clock rhythm genes,^19^ and its decrease may indicate weaker regulation in OA cartilage. Differential analysis of HomC between healthy and OA cartilages identified 454 OA-specific genes (Table S4) including *COL1A1* and *BGN* (Fig. 6C, D). We also identified 850 healthy specific genes (Table S4) including *WARS* and *ISG20* (Fig. 6E). GO enrichment analysis showed healthy specific genes in HomC enriched in cellular response to protein processing, immune system function, and maintenance of cellular homeostasis, indicative of their regulatory effect on cartilage homeostasis (Fig. 6F). However, OA-specific genes in HomC enriched in skeletal system development, degradation of the extracellular matrix, and ossification, implying their potential involvement in OA progression and pathological remodeling of the joint (Fig. 6F). These results indicated that HomC in OA cartilage decreased in number and were dysfunctional.

## Discussion

Here, we employed scRNA-seq to construct a single-cell transcriptomic atlas of chondrocytes in healthy human cartilage. We identified two HTC subpopulations with distinct functions and disparate terminal fates, namely HTC-1 and HTC-2. HTC-2 is involved in skeletal system development, which differentiate into PreFC and then FC. It is worth noting that HTC-1 specifically expresses genes related to apoptosis and programmed cell death and is the terminal of chondrocyte apoptosis trajectory at single-cell resolution. Importantly, we observed the expansion of the HTC-1 population in the cartilage of OA patients compared with the healthy individual. Compared with healthy cartilage, the OA-specific genes of HTC-1 showed weaker cellular response to stress and regulation of apoptosis, and are more likely to participate in vasculature development, degradation of the extracellular matrix, and ossification. These significant findings offer compelling clues indicating that an increased presence of HTC-1 and decreased chondrocyte apoptosis play pivotal roles in the pathogenesis of OA.

It is reported that the change in chondrocyte subpopulations and the cellular states may contribute to the occurrence of OA.^19^^,^^64^ Notably, the population size of ProFC in OA cartilage has significantly expanded compared with healthy cartilage. ProFC highly expressed cell cycle genes (*KIAA0101*, *STMN1*, and *MCM3*) and played an important role in extracellular matrix organization, collagen formation, and collagen fibril organization. Compared with ProFC in healthy cartilage, cellular response to cytokine stimulus and angiogenesis signals decreased in OA cartilage, while extracellular matrix organization, collagen fibril organization, and ossification increased in OA cartilage, indicating the dysfunction of ProFC. Interestingly, not only ProFC has significantly expanded, but also a new subset, namely ProFC-2 showed up in OA cartilage. Different from ProFC, ProFC-2 showed increased cytokine signaling, inflammatory response, and cellular responses to stimuli. Thus ProFC-2 is an OA cartilage-specific subpopulation and may contribute to the development of OA via inflammation.

HomC is known for its protective effects against cartilage degeneration and its pronounced expression of human circadian clock rhythm genes.^19^ Here, we successfully identified well-defined gene markers for HomC including *ATF3*, *DDIT3*, and *GDF15*,^21^^,^^65^ all of which have been linked to collagen synthesis, chondrocyte proliferation, and chondrocyte differentiation. Interestingly, our results showed that HomC in OA cartilage was significantly lower than those in healthy cartilage, providing an interesting insight into the molecular mechanism of OA.

In summary, this study provided a single-cell transcriptomic atlas of chondrocytes in healthy cartilage. In particular, we identified a novel THC subset, namely HTC-1, that specifically expressed genes associated with cell apoptosis and programmed cell death. We identified two main trajectories of chondrocytes, one of which differentiates into FC, while the other terminates in apoptosis. A comparison of chondrocyte subsets between healthy cartilage and OA cartilage showed that ProFC and HTC-1 populations expanded in OA patients, whereas the HomC population decreased. Interestingly, we also discovered an OA-specific ProFC subset, namely ProFC-2, which showed enhanced cytokine signaling and inflammatory response. Therefore, ProFC-2 may contribute to the development of OA via inflammation signaling. In short, our study promotes a better understanding of chondrocyte heterogeneity in articular cartilage and also provides a new insight into the mechanisms underlying the progression of OA.

## Author contributions

Wenfei Jin and Li Duan conceived and designed the project. Qi Zhang, Bin Zeng, and Guanming Chen performed the experiments. Changyuan Huang analyzed the scRNA-seq data with contributions from Wenhong Hou and Bo Zhou. Wenfei Jin, Ni Hong, and Guozhi Xiao supervised this project and interpreted the results. Changyuan Huang and Wenfei Jin wrote and revised the manuscript, with input from other authors. All authors read and approved the manuscript.

## Conflict of interests

All the authors declare no conflict of interests with the content of this manuscript. The authors declare no affiliation with or financial involvement in organizations or entities with a direct financial interest in the subject matter or materials discussed in the manuscript.

## Funding

This study was supported by the 10.13039/501100012166National Key R&D Program of China (No. 2021YFF1200900), 10.13039/501100001809National Natural Science Foundation of China (No. 32170646, 8197211), Guangdong Basic and Applied Basic Research Foundation (China) (No. 2023A1515011908 to N.H.), Key-Area Research and Development Program of Guangdong Province, China (No. 2023B1111020006), International Science and Technology Cooperation Program of Guangdong, China (No. 2021A0505030011), Shenzhen Innovation Committee of Science and Technology (Guangdong, China) (No. JCYJ20220818100401003), Shenzhen Science and Technology Program (Guangdong, China) (No. SGDX20201103095800003, GJHZ20200731095606019, JCYJ20220818100401003), and Shenzhen High-level Hospital Construction Fund (Guangdong, China) (No. 1801004).

## Data availability

The raw single-cell RNA sequencing data generated for this study can be accessed from the Genome Sequence Archive of the Beijing Institute of Genomics (BIG) Data center, BIG, Chinese Academy of Sciences, under accession number HRA004154 at http://bigd.big.ac.cn/gsa-human. The scRNA-seq data of chondrocytes Sw_data is available in GEO (GSE220243).

## Appendix A Supplementary data

Figure S1. Basic information about single-cell RNA-seq data and chondrocyte subsets. **(A, B)** Basic information of the data quality and quality control. **(C)** The dot plot showing the expression of cluster-specific markers in each chondrocyte subset. Dot size and color represent the fraction of cells with expression and the average normalized expression level, respectively. **(D)** The heatmap showing the expression of hypertrophic genes in chondrocyte subsets. **(E)** UMAP visualization of the expression of representative hypertrophic genes, FMOD (left) and EBF1 (right). **(F)** The ring graph showing the cell compositions of chondrocytes in healthy cartilage. **(G)** Inferred cell cycle states of each cell.

Figure S2. Gene ontology enrichment analysis of chondrocyte subset-specific genes.

Figure S3. The inferred cell–cell interaction patterns between chondrocyte subpopulations by different methods are consistent. **(A)** The heatmap of significant interactions between chondrocyte subsets inferred by CellPhoneDB. **(B)** The network plot showing the number of ligand–receptor interactions detected between chondrocyte subsets inferred by iTalk. **(C)** The network plot showing signal intensity sent out by each chondrocyte subset.

Figure S4. Cell–cell interaction signaling network among chondrocyte subsets. **(A–F)** Cell–cell contact signaling between chondrocyte subsets based on COLLAGEN signaling pathway (A), FN1 signaling pathway (B), THBS signaling pathway (C), LAMININ signaling pathway (D), TENASCIN signaling pathway (E), and HSPG signaling pathway (F) inferred by CellChat. The width and color of the line represent the strength of cell–cell interaction and signaling source, respectively. **(G)** The heatmap of the signaling pathway in chondrocyte subsets inferred by CellChat. The outgoing signaling patterns were based on accumulated weighted value (left panel) and incoming signaling patterns were based on accumulated weighted value (right panel). **(H)** The river plot showing the outgoing communication patterns (left) and the incoming communication patterns (right).

Figure S5. The pseudotime and trajectories of chondrocytes. **(A)** Trajectory inferred by scVelo. **(B)** Trajectory inferred by monocle3. **(C)** Pseudotime score of each chondrocyte. **(D)** The Venn diagram of genes between subpopulation-specific genes in Figure 1C and trajectory #1 associated genes in Figure 3G. **(E)** The Venn diagram of genes between subpopulation-specific genes in Figure 1C and trajectory #1 associated genes in Figure 3I.

Figure S6. Chondrocyte subsets and pseudotime of normal human cartilage in Sw_data. **(A)** UMAP visualization of the normal cartilage chondrocytes in Sw_data, colored by subset. **(B)** UMAP visualization of chondrocytes in each sample. **(C)** The dotplot of representative chondrocyte subset-specific genes. **(D)** The violin plots showing the expression levels of *CHI3L1* and *CHI3L2* in chondrocyte subsets of our data (from Fig. 4). **(E)** The violin plots showing the expression levels of *COL1A1* and *NBL1* in chondrocyte subsets of our data (from Fig. 4). **(F)** The pseudotime trajectories of chondrocytes inferred by monocle2. **(G)** The distribution of each chondrocyte subset along the trajectories.

Figure S7. Chondrocyte subsets in healthy cartilage and OA cartilage. **(A)** UMAP visualization of chondrocytes in healthy cartilage and OA cartilage. **(B)** UMAP visualization of chondrocyte subsets in each sample. **(C)** Correlation of gene expression between all cell subpopulations. **(D)** The Venn diagram of differentially expressed genes between Health, OA1, and OA2. **(E)** The violin plot of representative chondrocyte subset-specific genes. **(F)** Cell compositions of chondrocytes in healthy, OA1, and OA2 cartilage. **(G)** The bar plot displaying cell compositions of each chondrocyte subset based on cartilage sources. **(H)** The proportion of each chondrocyte subset to the total cells. OA, osteoarthritis.

Figure S8. Analyses of scRNA-seq data of normal cartilage and OA cartilage in Sw_data. **(A–C)** UMAP visualization of chondrocytes in normal and OA cartilage, colored by sample (A), colored by normal and OA (B), and colored by subpopulation (C). **(D)** UMAP visualization of chondrocytes of each sample, colored by subpopulation. **(E)** Comparison of the expression of chondrocyte subset-specific genes between normal cartilage and OA cartilage. **(F)** Comparison of fractions of chondrocyte subpopulations in normal and OA samples. **(G)** The violin plots of the expression levels of *COL11A1* and *HAPLN1* in chondrocyte subsets in our data (from Fig. 4). **(H)** Cell compositions in normal and OA cartilage (left), in each donor (middle), and each subset based on cell sources (right). **(I)** The proportion of cluster 5 (HomC) in normal and OA cartilage. **(J)** The proportion of cluster 10 (HTC-1) in normal and OA cartilage. OA, osteoarthritis; HomC, homeostatic chondrocytes; HTC, hypertrophic chondrocytes.

Figure S9. Comparison of ProFC and ProFC-2. **(A)** Highlighting of ProFC (left) and ProFC-2 (right) on the UMAP plot. **(B)** UMAP visualization of the expression of ProFC-specific genes and ProFC-2-specific genes. **(C)** Gene set enrichment analysis (GSEA) showed mitotic metaphase and anaphase were associated with ProFC-specific genes. **(D)** GSEA showed mitotic anaphase was associated with ProFC-2-specific genes. **(E)** The heatmap of the expression level of differentially expressed genes (DEGs) between ProFC and ProFC-2. **(F)** The violin plots of representative ProFC-specific genes in ProFC and ProFC-2. **(G)** The violin plots of representative ProFC-2-specific genes in ProFC and ProFC-2. **(H)** Gene ontology enrichment analysis of ProFC-specific genes and ProFC-2-specific genes. ProFC, proliferate fibrochondrocytes.
