# Supplementary material for: Single-cell transcriptomic analysis of chondrocytes in cartilage and pathogenesis of osteoarthritis
Source: Genes Dis. 2024 Feb 2;12(2):101241. doi: 10.1016/j.gendis.2024.101241 (PMC11697194; doi:10.1016/j.gendis.2024.101241)
Supplement: Multimedia component 1 [file mmc1.pdf]

FigS1

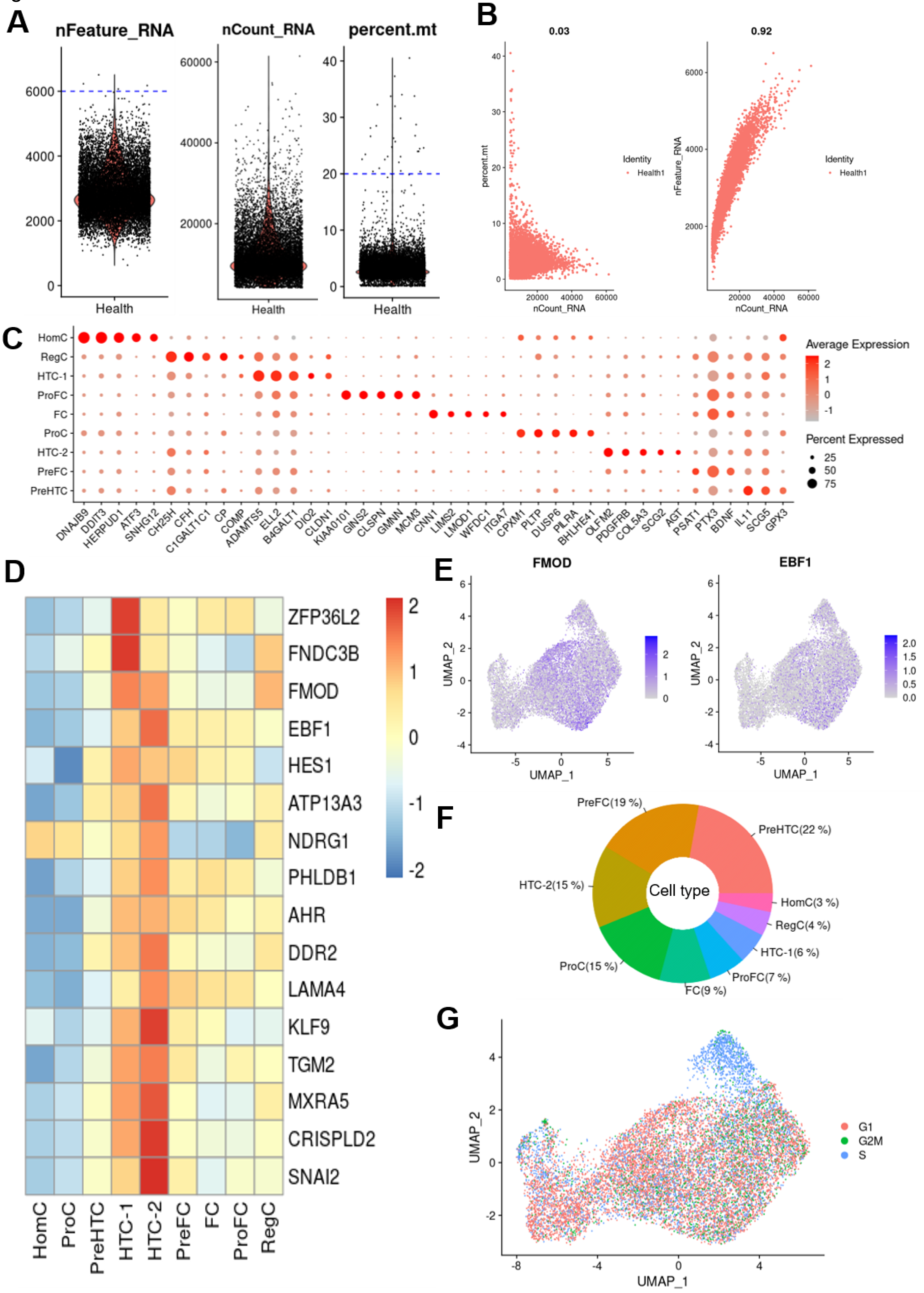
A
nFeature_RNA
nCount_RNA
percent.mt
Health
B
0.03
0.92
percent.mt
nCount_RNA
nFeature_RNA
Identity
Health1
C
HomC
RegC
HTC-1
ProFC
FC
ProC
HTC-2
PreFC
PreHTC
DNAJB9 DDIT3 HERPUD1 ATF3 SNHG12 CH25H CFH C1GALT1C1 CP COMP ADAMTS5 ELL2 B4GALT1 DIO2 CLDN1 KIAA0101 GINS2 CLSPN GMNN MCM3 CNN1 LIMS2 LMOD1 WFDC1 ITGA7 CPXM1 PLTP DUSP6 PILRA BHLHE41 OLFM2 PDGFRB COL5A3 SCG2 AGT PSAT1 PTX3 BDNF IL11 SCG5 GPX3
Average Expression
Percent Expressed
D
ZFP36L2
FNDC3B
FMOD
EBF1
HES1
ATP13A3
NDRG1
PHLDB1
AHR
DDR2
LAMA4
KLF9
TGM2
MXRA5
CRISPLD2
SNAI2
HomC ProC PreHTC HTC-1 HTC-2 PreFC FC ProFC RegC
E
FMOD
EBF1
UMAP_1
UMAP_2
F
Cell type
PreFC(19 %)
PreHTC(22 %)
HTC-2(15 %)
HomC(3 %)
RegC(4 %)
HTC-1(6 %)
ProC(15 %)
ProFC(7 %)
FC(9 %)
G
G1
G2M
S
UMAP_1
UMAP_2

FigS2

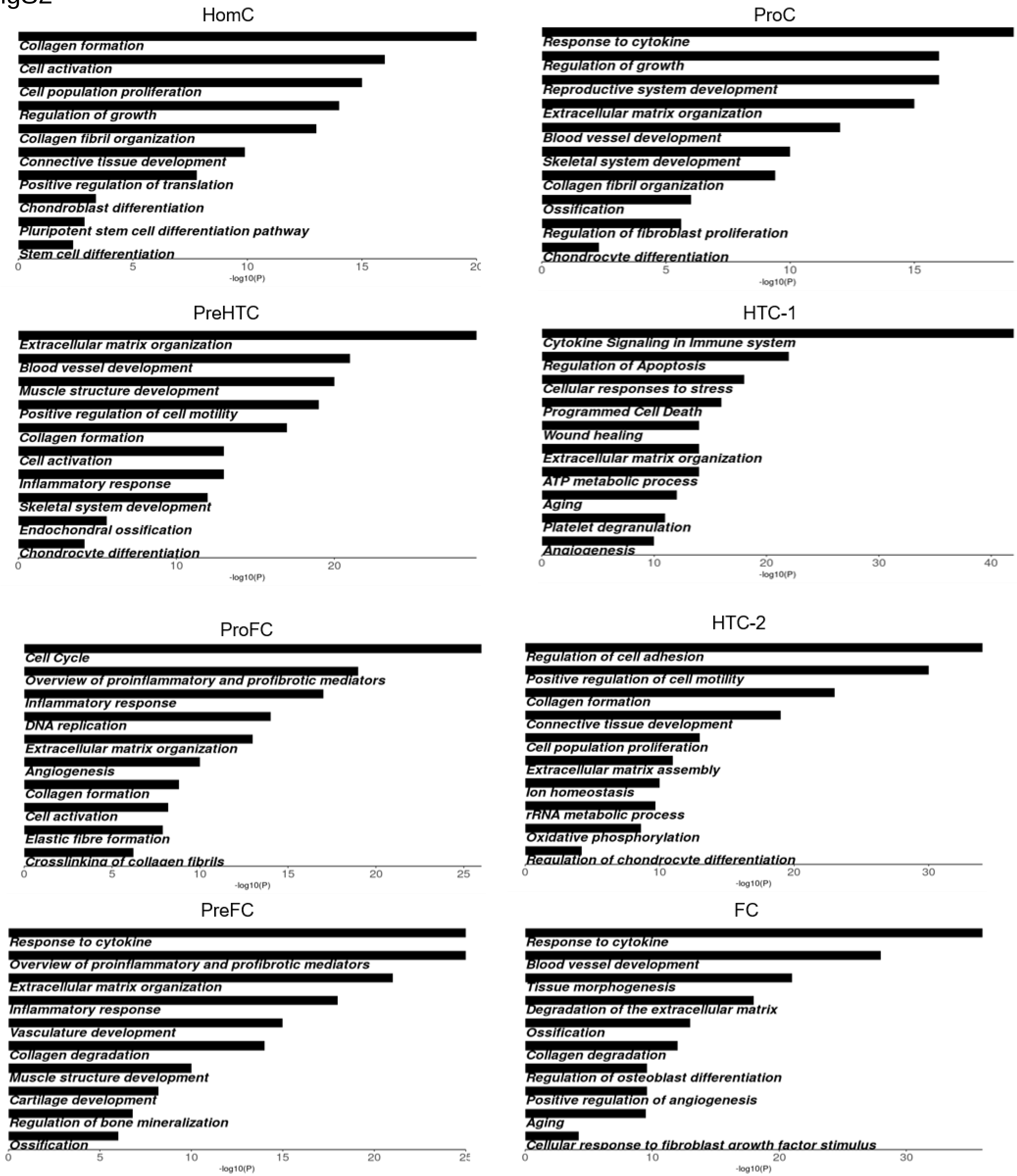
HomC
Collagen formation
Cell activation
Cell population proliferation
Regulation of growth
Collagen fibril organization
Connective tissue development
Positive regulation of translation
Chondroblast differentiation
Pluripotent stem cell differentiation pathway
Stem cell differentiation
0 5 10 15 20
-log10(P)
ProC
Response to cytokine
Regulation of growth
Reproductive system development
Extracellular matrix organization
Blood vessel development
Skeletal system development
Collagen fibril organization
Ossification
Regulation of fibroblast proliferation
Chondrocyte differentiation
0 5 10 15
-log10(P)
PreHTC
Extracellular matrix organization
Blood vessel development
Muscle structure development
Positive regulation of cell motility
Collagen formation
Cell activation
Inflammatory response
Skeletal system development
Endochondral ossification
Chondrocyte differentiation
0 10 20
-log10(P)
HTC-1
Cytokine Signaling in Immune system
Regulation of Apoptosis
Cellular responses to stress
Programmed Cell Death
Wound healing
Extracellular matrix organization
ATP metabolic process
Aging
Platelet degranulation
Angiogenesis
0 10 20 30 40
-log10(P)
ProFC
Cell Cycle
Overview of proinflammatory and profibrotic mediators
Inflammatory response
DNA replication
Extracellular matrix organization
Angiogenesis
Collagen formation
Cell activation
Elastic fibre formation
Crosslinking of collagen fibrils
0 5 10 15 20 25
-log10(P)
HTC-2
Regulation of cell adhesion
Positive regulation of cell motility
Collagen formation
Connective tissue development
Cell population proliferation
Extracellular matrix assembly
Ion homeostasis
rRNA metabolic process
Oxidative phosphorylation
Regulation of chondrocyte differentiation
0 10 20 30
-log10(P)
PreFC
Response to cytokine
Overview of proinflammatory and profibrotic mediators
Extracellular matrix organization
Inflammatory response
Vasculature development
Collagen degradation
Muscle structure development
Cartilage development
Regulation of bone mineralization
Ossification
0 5 10 15 20 25
-log10(P)
FC
Response to cytokine
Blood vessel development
Tissue morphogenesis
Degradation of the extracellular matrix
Ossification
Collagen degradation
Regulation of osteoblast differentiation
Positive regulation of angiogenesis
Aging
Cellular response to fibroblast growth factor stimulus
0 10 20 30
-log10(P)

FigS3

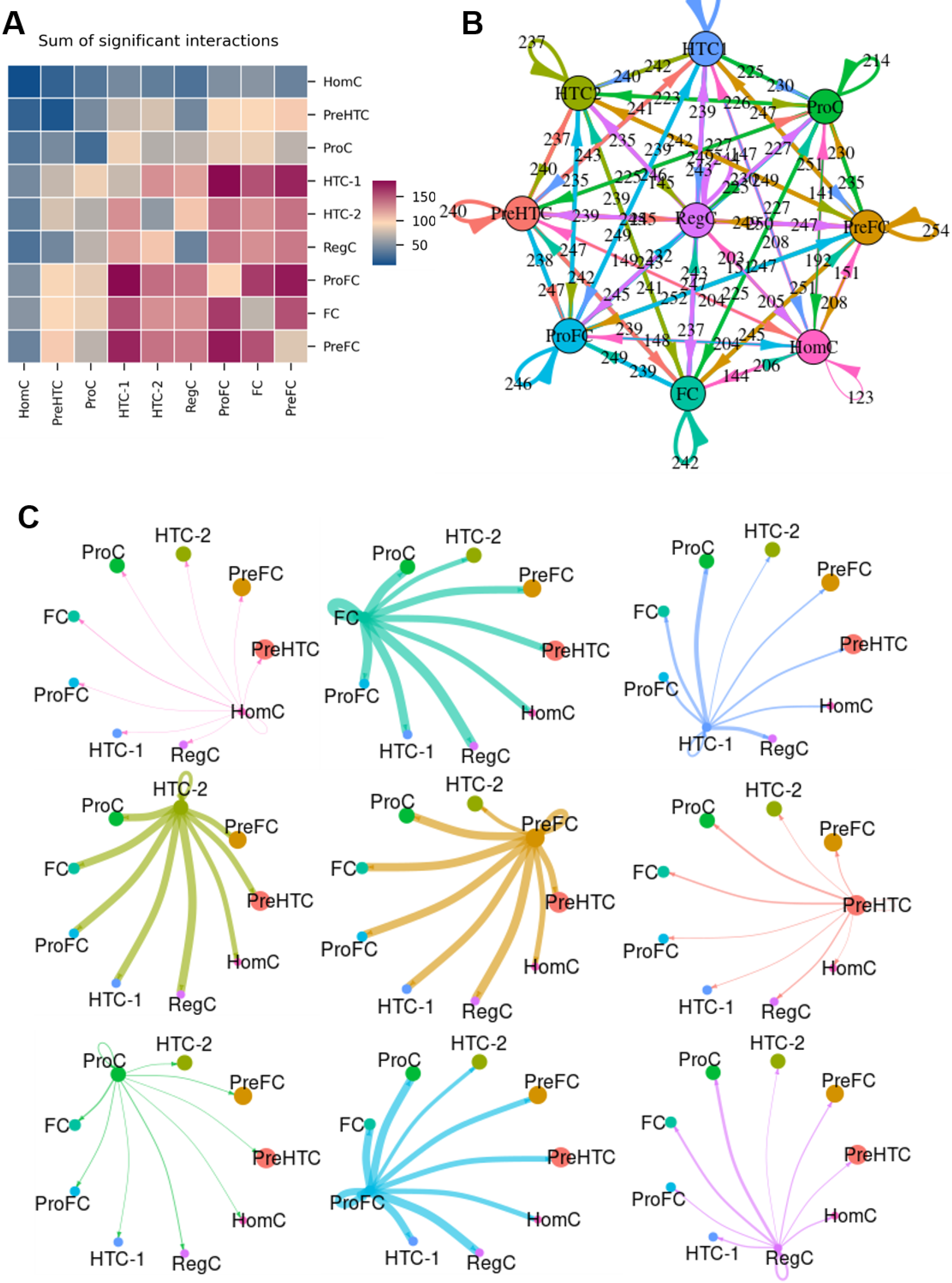

A
Sum of significant interactions
HomC
PreHTC
ProC
HTC-1
HTC-2
RegC
ProFC
FC
PreFC
150
100
50
B
HTC1
HTC2
ProC
PreHTC
RegC
PreFC
ProFC
HomC
FC
C
HomC
FC
HTC-1
HTC-2
PreFC
PreHTC
ProC
ProFC
RegC

FigS4

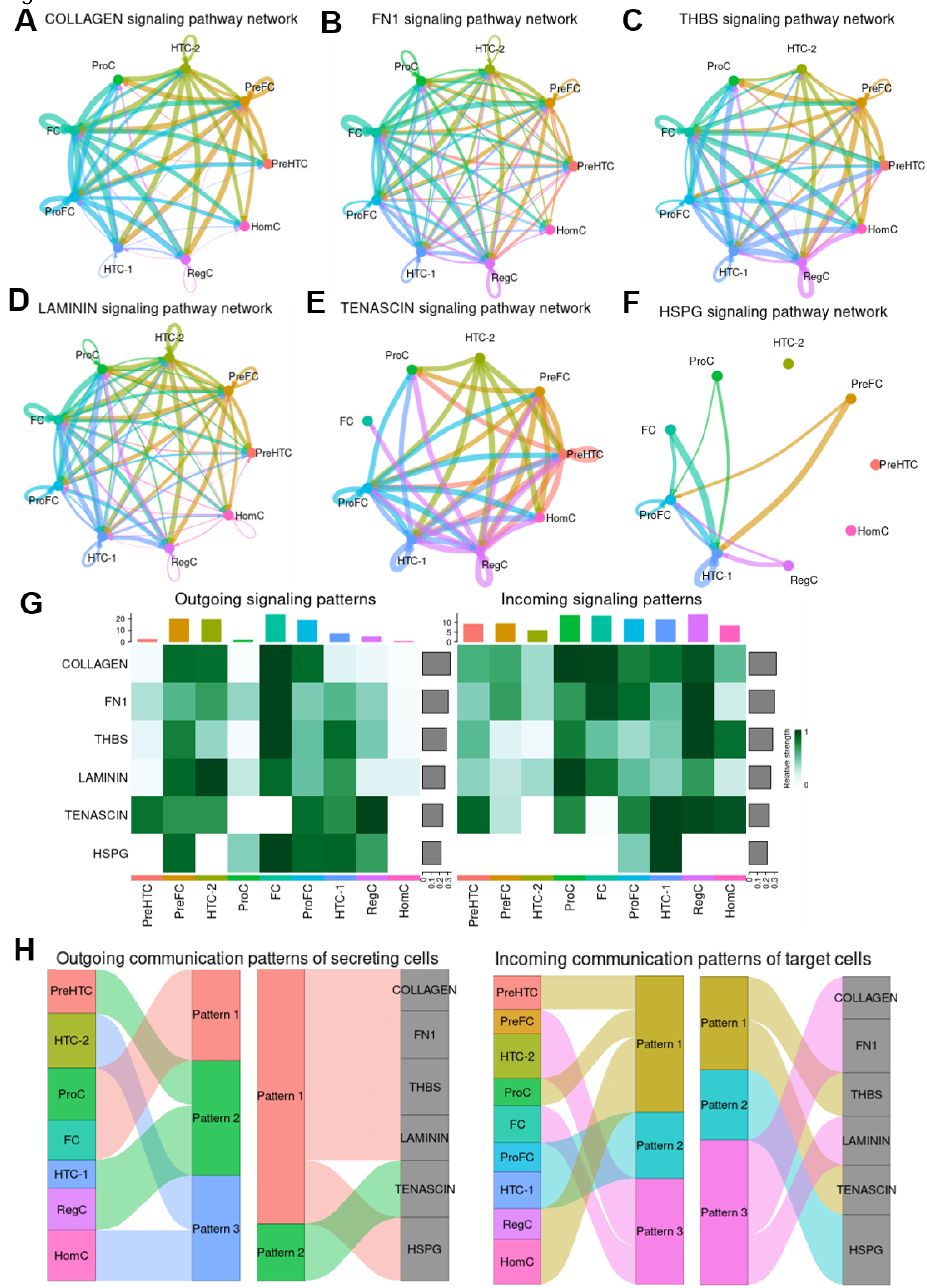

A
COLLAGEN signaling pathway network
B
FN1 signaling pathway network
C
THBS signaling pathway network
D
LAMININ signaling pathway network
E
TENASCIN signaling pathway network
F
HSPG signaling pathway network
HTC-2
ProC
PreFC
FC
PreHTC
ProFC
HomC
HTC-1
RegC
G
Outgoing signaling patterns
Incoming signaling patterns
COLLAGEN
FN1
THBS
LAMININ
TENASCIN
HSPG
Relative strength
H
Outgoing communication patterns of secreting cells
Incoming communication patterns of target cells
Pattern 1
Pattern 2
Pattern 3

FigS5

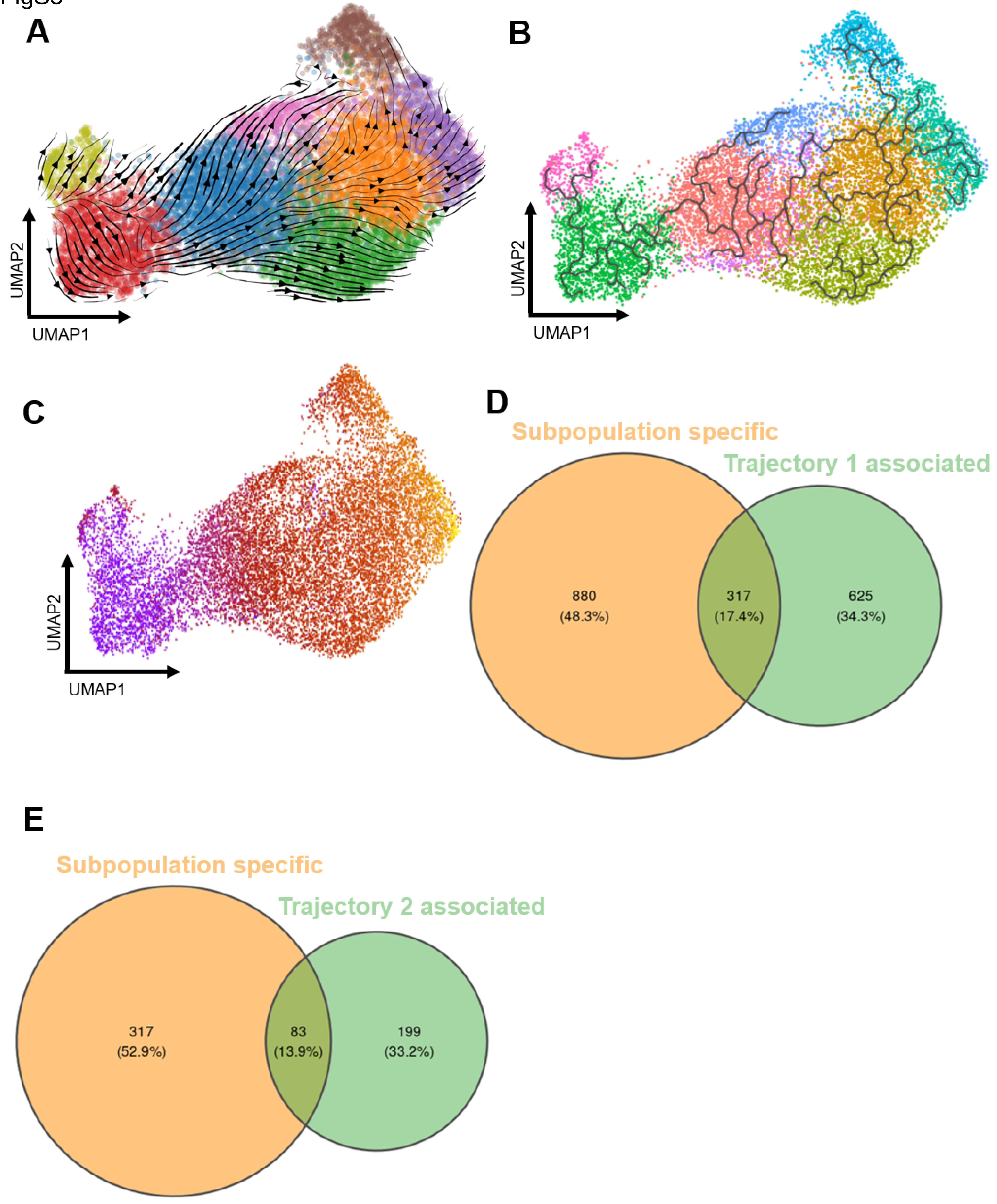

A
UMAP2
UMAP1
B
UMAP2
UMAP1
C
UMAP2
UMAP1
D
Subpopulation specific
Trajectory 1 associated
880
(48.3%)
317
(17.4%)
625
(34.3%)
E
Subpopulation specific
Trajectory 2 associated
317
(52.9%)
83
(13.9%)
199
(33.2%)

FigS6

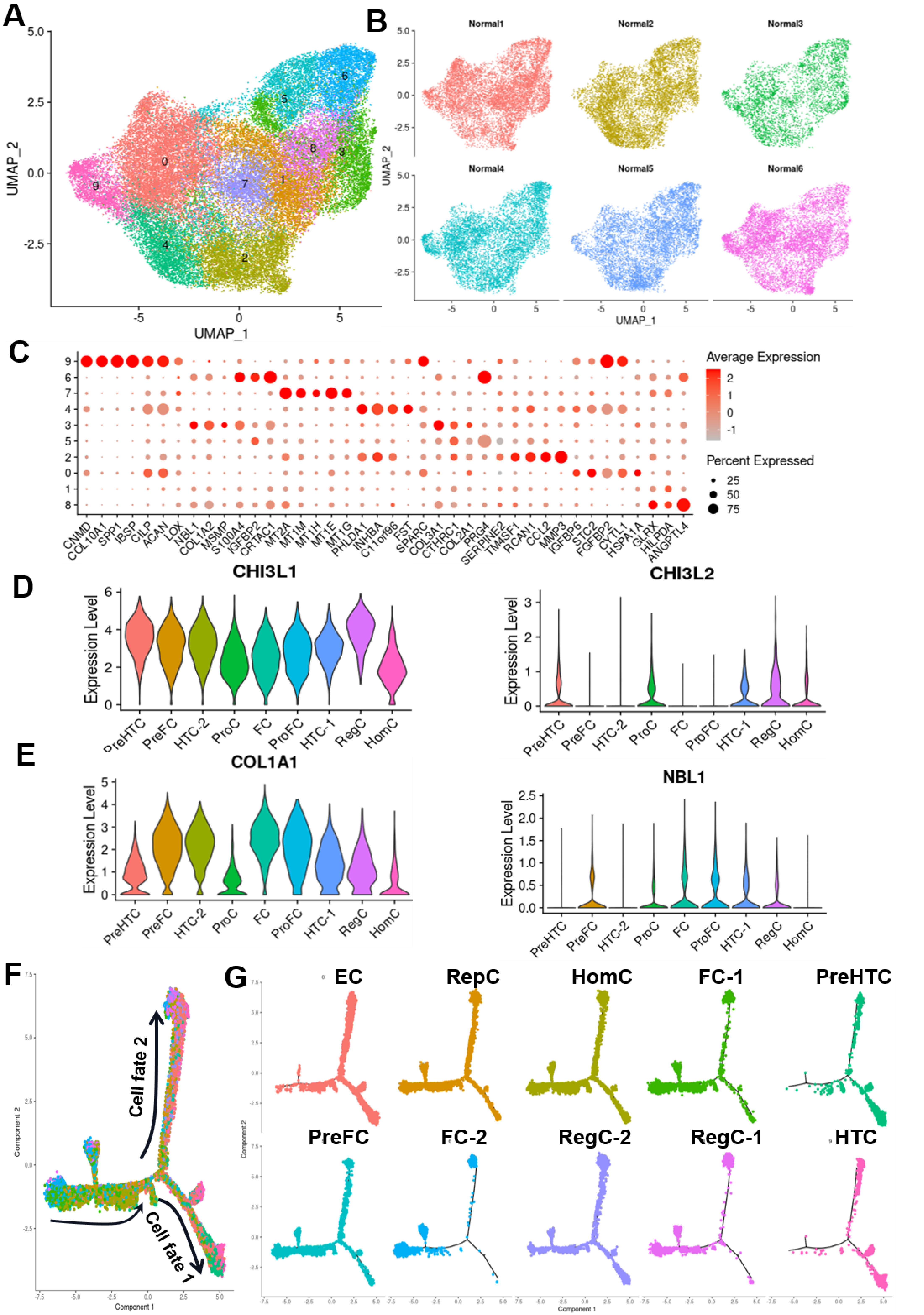

A
UMAP_1
UMAP_2
B
Normal1
Normal2
Normal3
Normal4
Normal5
Normal6
C
Average Expression
Percent Expressed
CNMD COL10A1 SPP1 IBSP CILP ACAN LOX NBL1 COL1A2 MSMP S100A4 IGFBP2 CRTAC1 MT2A MT1M MT1H MT1E MT1G PHLDA1 INHBA C11orf96 FST SPARC COL3A1 CTHRC1 COL2A1 PRG4 SERPINE2 TM4SF1 RCAN1 CCL2 MMP3 IGFBP6 STC2 FGFBP2 CYTL1 HSPA1A GLRX HILPDA ANGPTL4
D
CHI3L1
CHI3L2
Expression Level
PreHTC PreFC HTC-2 ProC FC ProFC HTC-1 RegC HomC
E
COL1A1
NBL1
F
Cell fate 2
Cell fate 1
Component 1
Component 2
G
EC
RepC
HomC
FC-1
PreHTC
PreFC
FC-2
RegC-2
RegC-1
HTC

FigS7

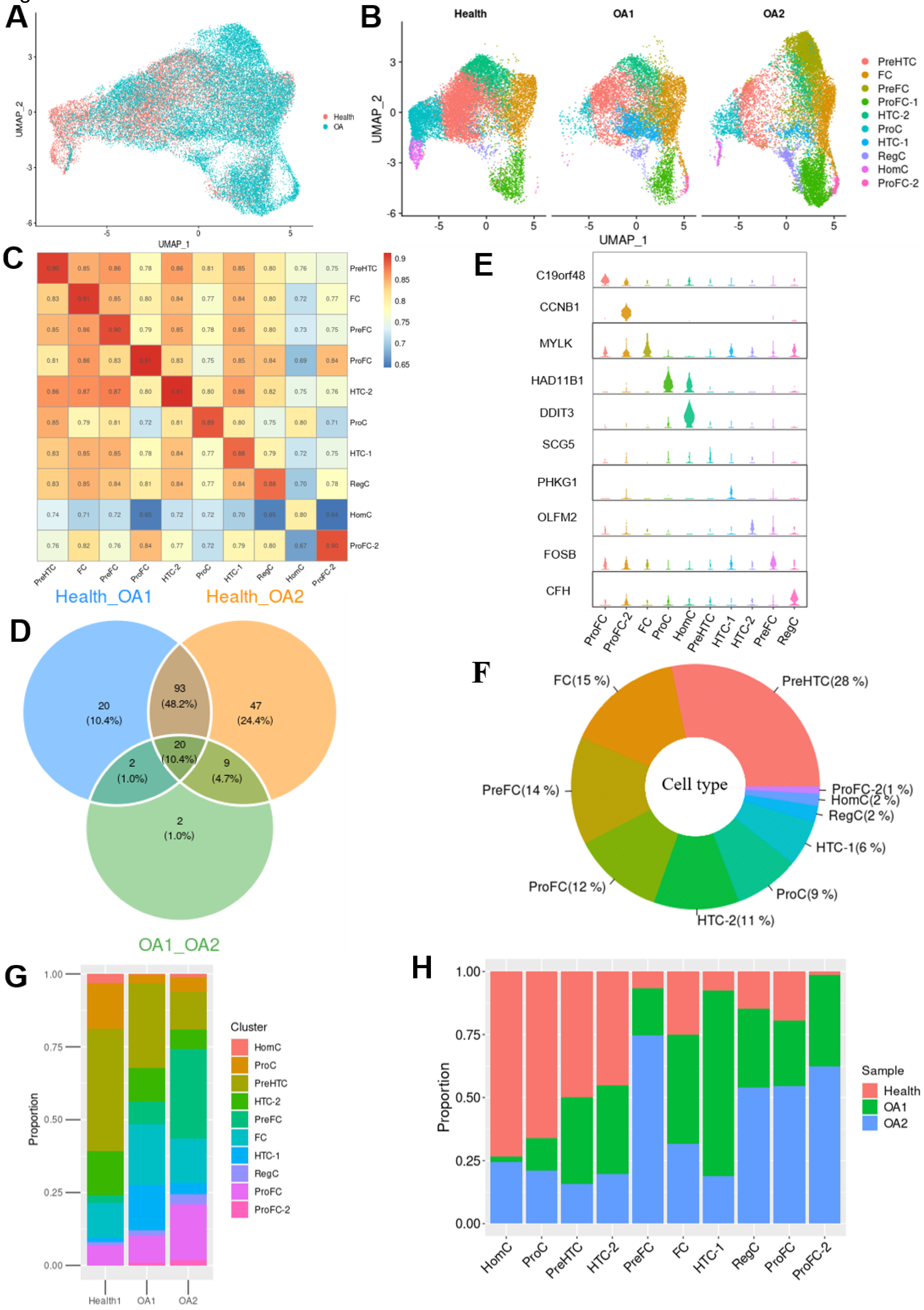
A
UMAP_2
UMAP_1
Health
OA
B
Health
OA1
OA2
UMAP_2
UMAP_1
PreHTC
FC
PreFC
ProFC-1
HTC-2
ProC
HTC-1
RegC
HomC
ProFC-2
C
0.90 0.85 0.86 0.78 0.86 0.81 0.85 0.80 0.76 0.75 PreHTC
0.83 0.91 0.85 0.80 0.84 0.77 0.84 0.80 0.72 0.77 FC
0.85 0.86 0.90 0.79 0.85 0.78 0.85 0.80 0.73 0.75 PreFC
0.81 0.86 0.83 0.91 0.83 0.75 0.85 0.84 0.69 0.84 ProFC
0.86 0.87 0.87 0.80 0.91 0.80 0.86 0.82 0.75 0.76 HTC-2
0.85 0.79 0.81 0.72 0.81 0.89 0.80 0.75 0.80 0.71 ProC
0.83 0.85 0.85 0.78 0.84 0.77 0.88 0.79 0.72 0.75 HTC-1
0.83 0.85 0.84 0.81 0.84 0.77 0.84 0.88 0.70 0.78 RegC
0.74 0.71 0.72 0.65 0.72 0.72 0.70 0.65 0.80 0.64 HomC
0.76 0.82 0.76 0.84 0.77 0.72 0.79 0.80 0.67 0.90 ProFC-2
PreHTC FC PreFC ProFC HTC-2 ProC HTC-1 RegC HomC ProFC-2
D
Health_OA1
Health_OA2
OA1_OA2
20 (10.4%)
93 (48.2%)
47 (24.4%)
20 (10.4%)
2 (1.0%)
9 (4.7%)
2 (1.0%)
E
C19orf48
CCNB1
MYLK
HAD11B1
DDIT3
SCG5
PHKG1
OLFM2
FOSB
CFH
ProFC ProFC-2 FC ProC HomC PreHTC HTC-1 HTC-2 PreFC RegC
F
Cell type
PreHTC(28 %)
FC(15 %)
PreFC(14 %)
ProFC(12 %)
HTC-2(11 %)
ProC(9 %)
HTC-1(6 %)
RegC(2 %)
HomC(2 %)
ProFC-2(1 %)
G
Proportion
Cluster
HomC
ProC
PreHTC
HTC-2
PreFC
FC
HTC-1
RegC
ProFC
ProFC-2
Health1
OA1
OA2
H
Proportion
Sample
Health
OA1
OA2
HomC ProC PreHTC HTC-2 PreFC FC HTC-1 RegC ProFC ProFC-2

FigS8

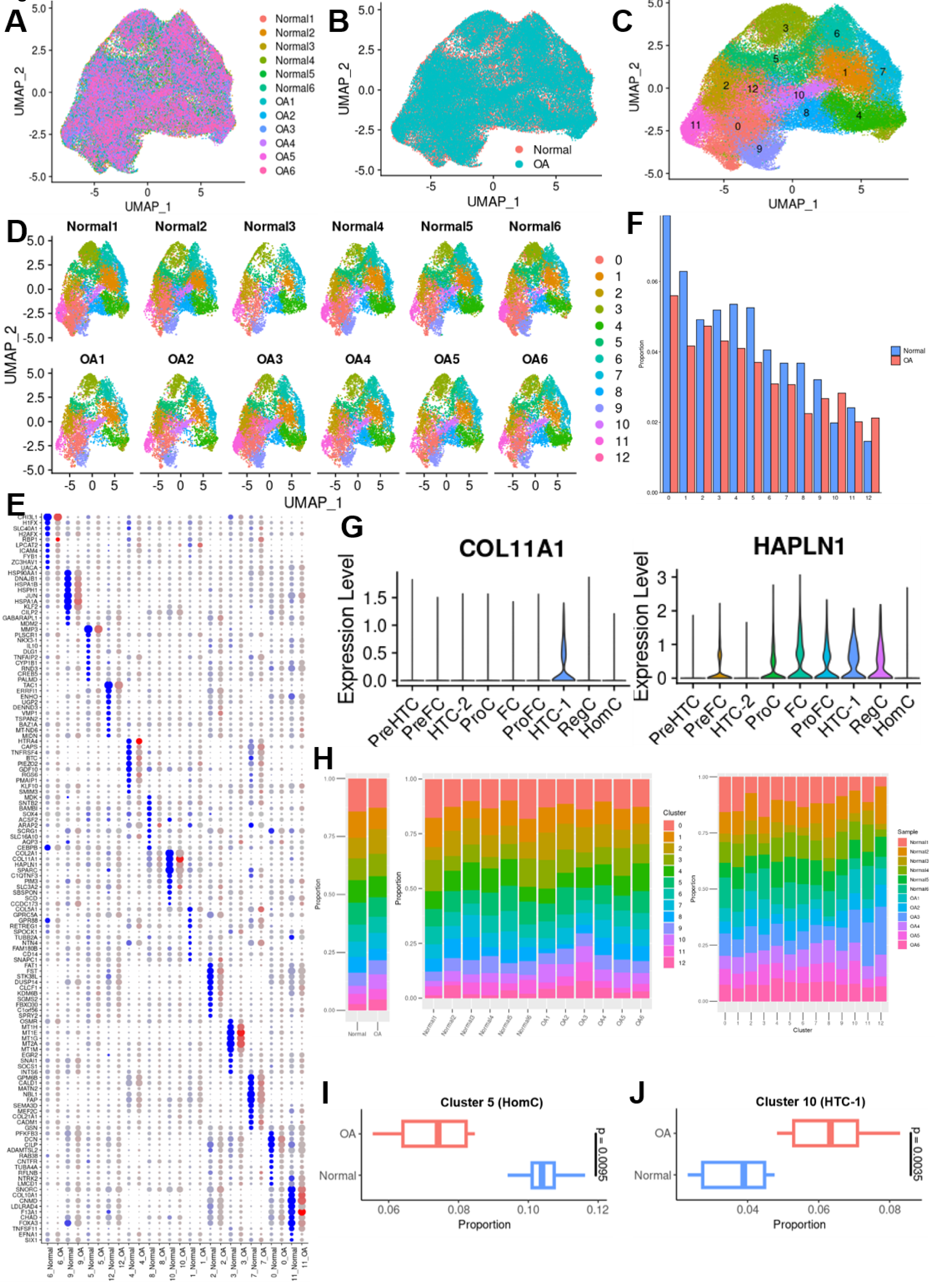

A
B
C
D
E
F
G
H
I
J
UMAP_1
UMAP_2
Normal1
Normal2
Normal3
Normal4
Normal5
Normal6
OA1
OA2
OA3
OA4
OA5
OA6
Normal
OA
COL11A1
HAPLN1
Expression Level
PreHTC
PreFC
HTC-2
ProC
FC
ProFC
HTC-1
RegC
HomC
Proportion
Cluster
Sample
Cluster 5 (HomC)
Cluster 10 (HTC-1)
p = 0.0095
p = 0.0035

FigS9

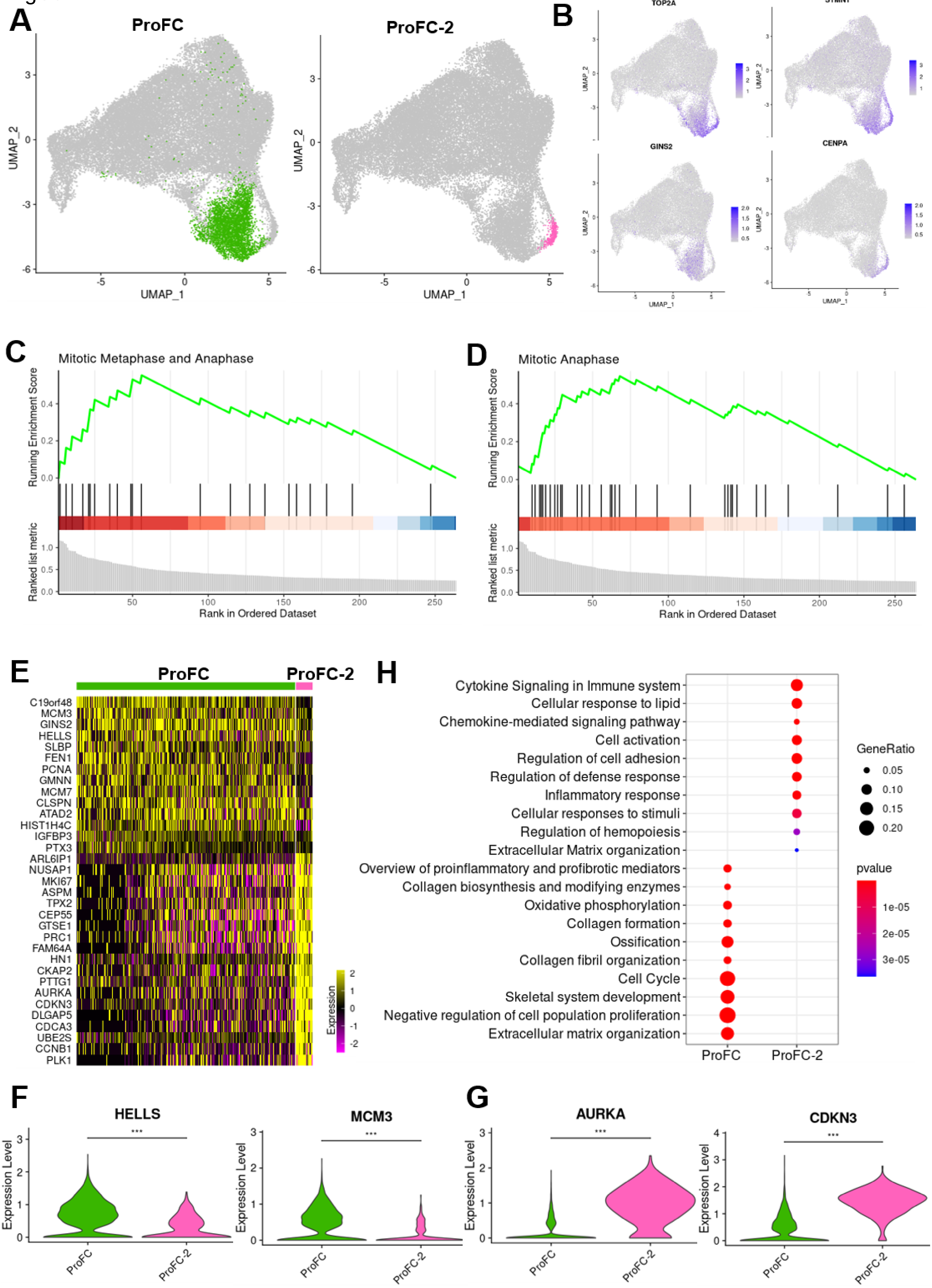

A
ProFC
ProFC-2
UMAP_1
UMAP_2
B
TOP2A
STMN1
GINS2
CENPA
C
Mitotic Metaphase and Anaphase
Running Enrichment Score
Ranked list metric
Rank in Ordered Dataset
D
Mitotic Anaphase
E
ProFC
ProFC-2
C19orf48
MCM3
GINS2
HELLS
SLBP
FEN1
PCNA
GMNN
MCM7
CLSPN
ATAD2
HIST1H4C
IGFBP3
PTX3
ARL6IP1
NUSAP1
MKI67
ASPM
TPX2
CEP55
GTSE1
PRC1
FAM64A
HN1
CKAP2
PTTG1
AURKA
CDKN3
DLGAP5
CDCA3
UBE2S
CCNB1
PLK1
Expression
H
Cytokine Signaling in Immune system
Cellular response to lipid
Chemokine-mediated signaling pathway
Cell activation
Regulation of cell adhesion
Regulation of defense response
Inflammatory response
Cellular responses to stimuli
Regulation of hemopoiesis
Extracellular Matrix organization
Overview of proinflammatory and profibrotic mediators
Collagen biosynthesis and modifying enzymes
Oxidative phosphorylation
Collagen formation
Ossification
Collagen fibril organization
Cell Cycle
Skeletal system development
Negative regulation of cell population proliferation
Extracellular matrix organization
ProFC
ProFC-2
GeneRatio
0.05
0.10
0.15
0.20
pvalue
1e-05
2e-05
3e-05
F
HELLS
MCM3
Expression Level
G
AURKA
CDKN3
