# Supplementary material for: Single-cell transcriptomic analysis of chondrocytes in cartilage and pathogenesis of osteoarthritis
Source: Genes Dis. 2024 Feb 2;12(2):101241. doi: 10.1016/j.gendis.2024.101241 (PMC11697194; doi:10.1016/j.gendis.2024.101241)
Supplement: Multimedia component 2 [file mmc2.docx]

**Table S1** Individual information of samples.

| Sample ID | Characteristics | Species | Gender | Age | Sequencing instrument | Number of cells | Median unique molecular index per cell | Median genes per cell |
| --- | --- | --- | --- | --- | --- | --- | --- | --- |
| Health | Chondrocytes from healthy human knee cartilage | Homo sapiens | Female | 59 | Illumina NovaSeq 6000 | 13,397 | 10,606 | 2753 |
| OA1 | Chondrocytes from human knee cartilage with osteoarthritis | Homo sapiens | Female | 67 | Illumina NovaSeq 6000 | 8844 | 16,191 | 3537 |
| OA2 | Chondrocytes from human knee cartilage with osteoarthritis | Homo sapiens | Female | 60 | Illumina NovaSeq 6000 | 12,784 | 13,046 | 2890 |
